# Big data ordination towards intensive care event count cases using fast computing GLLVMS

**DOI:** 10.1186/s12874-022-01538-4

**Published:** 2022-03-21

**Authors:** Rezzy Eko Caraka, Rung-Ching Chen, Su-Wen Huang, Shyue-Yow Chiou, Prana Ugiana Gio, Bens Pardamean

**Affiliations:** 1 Executive Secretariat, National Research and Innovation Agency (BRIN), DKI Jakarta, 10340 Indonesia; 2 Department of Information Management, College of Informatics, Chaoyang University of Technology, Taichung City, 41349 Taiwan; 3 Taichung Veterans General Hospital, Taichung City, 40705 Taiwan; 4 Department of Mathematics, Universitas Sumatera Utara, Medan, 20155 Indonesia; 5 Bioinformatics and Data Science Research Center, Bina Nusantara University, DKI Jakarta, 11480 Indonesia; 6 Computer Science Department, Bina Nusantara University, DKI Jakarta, 11480 Indonesia

**Keywords:** GLLVM, Fast Computing, Laplace Approximation, Variational approximation, Ordination

## Abstract

**Background:**

In heart data mining and machine learning, dimension reduction is needed to remove multicollinearity. Meanwhile, it has been proven to improve the interpretation of the parameter model. In addition, dimension reduction can also increase the time of computing in high dimensional data.

**Methods:**

In this paper, we perform high dimensional ordination towards event counts in intensive care hospital for Emergency Department (ED 1), First Intensive Care Unit (ICU1), Second Intensive Care Unit (ICU2), Respiratory Care Intensive Care Unit (RICU), Surgical Intensive Care Unit (SICU), Subacute Respiratory Care Unit (RCC), Trauma and Neurosurgery Intensive Care Unit (TNCU), Neonatal Intensive Care Unit (NICU) which use the Generalized Linear Latent Variable Models (GLLVM’s).

**Results:**

During the analysis, we measure the performance and calculate the time computing of GLLVM by employing variational approximation and Laplace approximation, and compare the different distributions, including Negative Binomial, Poisson, Gaussian, ZIP, and Tweedie, respectively. GLLVMs (Generalized Linear Latent Variable Models), an extended version of GLMs (Generalized Linear Models) with latent variables, have fast computing time. The major challenge in latent variable modelling is that the function $f\left(\varTheta \right)=\int f\left(u\varTheta \right)h\left(u\right)du$ is not trivial to solve since the marginal likelihood involves integration over the latent variable *u*.

**Conclusions:**

In a nutshell, GLLVMs lead as the best performance reaching the variance of 98% comparing other methods. We get the best model negative binomial and Variational approximation, which provides the best accuracy by accuracy value of AIC, AICc, and BIC. In a nutshell, our best model is GLLVM-VA Negative Binomial with AIC 7144.07 and GLLVM-LA Negative Binomial with AIC 6955.922.

**Supplementary Information:**

The online version contains supplementary material available at 10.1186/s12874-022-01538-4.

## Background

Big data is collecting massive data and is more complex, especially from new data sources [1]. The data set is large enough, so that software for traditional data processors is not good enough to manage it. Still, this massive amount of data can be used to overcome a variety of business problems that previously could not be solved for the decision-making [2]. The most straightforward and obvious explanation is that Big Data collects and uses various sources to provide important information. Big Data is also a concept of collecting, analysing, and understanding many data on a comprehensive range of activities. Big Data is profitable for the hospital service system. One of the classic problems is that there are excessively many staff or too few staff, so the hospital will risk incurring additional costs than they should. Not mainly that, hospitals that lose staff will also expose the quality and performance of the performed services.

If a few teams handle many patients, this will directly impact the services. These patients will be of poor quality and unsatisfactory. The primary key to implementing hospital orientation is the patient. Then, patient satisfaction is the success of a hospital in managing health care services. Customer satisfaction is an abstract thing, and the results are very varied.

However, perceptions depend on each person and tend to be different. The availability of medical personnel with high knowledge and skills is essential for patients choosing a health service to help them recover from the disease. The core business of the hospital is to provide health services. A good hospital can offer professional medical personnel and provide the best facilities and an excellent patient-care system [3]. At the same time, monitoring patient clinical status is essential, particularly in intensive care units (ICUs) [4]. During that time, the teleporter plays the role of “facilitator” and “supporter”. It is one of the medical team’s valuable members and the connection window between the unit and the department.

The transmission staff is responsible for assisting the patient’s medical treatment or acting as a helper for the family to care for the patient. It must have sufficient resilience to respond to the emergency that may occur, and the transmission process must strictly follow the actual transfer and relevant safety rules. The mastery, accuracy, and completeness of the delivery service time relate to the smooth connection of medical services, so it must have a certain degree of job sensitivity and excellent communication skills. Furthermore, with patients’ increasing needs and desires in obtaining the best services, it is necessary to do the proper planning, especially in the intensive care centre room.

The most crucial point is to place appropriate medical personnel in the intensive care centre. If the placement of medical staff is proper, hospital services will be better, and patients will be treated faster. Then another thing is to provide training to improve the work of medical personnel. If the human resources are of high quality and in line with company expectations, the company has high competitiveness. Therefore, the products and services produced high quality.

Intensive care units (ICUs) of university hospitals and advanced medical centres are indispensable for providing critical and intensive care for patients who have undergone major surgery or have received emergency care. Hospitals can obtain higher revenue from national insurance by a short admission in the ICU than by access to other hospital departments. Intensive care units are the foremost part and are very important in the hospital. Intensive care units act as the main entry gate for emergency patients and patients with mild conditions. Good or bad service in the intensive care unit will give an overall impression of hospital services. Analysing the number of events in the ICU is also essential to study. The cost estimation and a profit and loss analysis are necessary for the health care field [5].

A significant part of this work is to decide whether ICU care procedures can improve results for those identified as frailty. The instances of processes that may differ in the little incorporate wholesome help and sedation rehearse the force of assembly/restoration. In other words, an analysis of the number of medical personnel needs is essential in the ICU room; a first aid kit is needed quickly and temporarily to give a person suffering from an injury or sudden illness. First aid’s fundamental objective is to provide care and health services that benefit these people in preparation for further treatments.

An emergency is a condition related to a disease or other life-threatening illnesses. In contrast, a crisis is a sudden and unforeseen condition with an immediate or urgent need [6]. The emergency room’s operational nature must be fast, precise, and not limited by the time [7]. At the same time, we need to be concerned that the ideal performance of the emergency room is highly dependent on human resources and proper work procedures. Moreover, the supporting examination facilities can support the diagnostic process. The adequate drug support and medical consumables clear patients in and out, ready the operating room, and ambulance transport support that focuses on patient safety.

Big Data Analysis offers an excellent opportunity to improve strategic unit management and handle concrete clinical cases [8–10]. Moreover, different biomedical and medicinal services devices produce a primary information field measure [11]. We must think about and evaluate what can be accomplished by utilising this information field [12]. The problem is hard to select large-dimensional data; many attributes and causing some algorithms to be complex to get good performance. Therefore, the solution offered is to do feature selection or dimension reduction by using PCA [13–15], K-means [2], CCA [16], Factor analysis [17–19], eXtreme Gradient Boosting (XGBoost) [20–22] Bayesian [23–25].

Nowadays, there is challenging to measure statistical parameters in vast data sets, and most traditional statistical methods cannot handle high dimensional data and large numbers of parameters [24, 26–29]. This situation additionally typically mirrored the contemporary impediments of computing. In short, this research will get an ordinance of intensive care hospital rooms so that we can use it to calculate and predict how many patients are expected to be in the room daily and hourly. The remainder of the paper is organised as follows. Section 2 explains the methods. Section 3 presents the application of a high dimension. Section 4 presents the results and analysis. Finally, conclusions and future research directions are indicated in Sect. 4.

## Methods

### Generalized Linear Models

In its development, the modelling of count data led to Generalized Linear Models (GLMs) [8]. GLMs are generalisations of classical regression models or OLS regression. Analytical methods for data do not meet the assumption of a normal distribution [30]. The classical linear model is widely used in statistics or straight-line equation [31]. The traditional linear models were commonly used in statistics, especially for modelling field environmental problems [32]. The simplest classical non-linear model is defined in Eq. (1).

1 $$y=f\left(x,\beta\right)+\epsilon$$

Where *y* is the dependent variable whose value depends on the independent variable *x, β* which are unknown parameters in the model. At the same time, *ε* is a random variable that differs from the actual value of *y* with its estimated value. The random variable *ε* is assumed to follow the Normal distribution $\left(0,{\varvec{\sigma }}^{2}\right)$. The development of the linear model was very rapid after discovering the normal distribution until the beginning of the 19th century that [33] published his research in agriculture using an experimental design. Simple GLMs are developing a classic linear model with many predictors or multiple linear regression [34]. The least-square method by Gauss remains the basis for estimating model parameters. The assumptions on LMs also carry over to GLMs is$\epsilon$ follows the Normal distribution $\left(0,{\sigma }^{2}\right)$. The predictor does not need continuous. Category predictors also underlie Fisher’s research in experimental design. Under the auspices of the normal distribution assumption, linear models can be written in general, or the general term defines GLM as in Eq. (2).

2 $$Y_nG_n=X_nBH_n+Z_n\backslash\mathrm{varTheta}Q_n+E_n$$

The model in Eq. (2) represents the GLMs for various linear models. They are linear regression (simple or multiple), multivariate regression, analysis of variance (ANOVA), multivariate analysis of variance (MANOVA), linear mixed models, analysis of variance-covariance (ANCOVA), multivariate analysis of covariance (MANCOVA), response surface, or growth curve model. In addition to the least square, parameter estimation can be obtained using the maximum likelihood, shrinkage estimation, stein-rule estimation method up to Bayes estimation approach. Operationalism means that scientific theories should be defined observably, namely observation or observational or experimental procedures.

In early 20th century, there have been many books published like “foundation of Statistics, the foundation of Ethics, foundation of justice, foundation of fairness” All of these books are studying foundational principles for their subjects, to enable deductive logic to justify necessary propositions of these areas [35–37]. Thus, in deductive logic if the general propositions are true, their logical statements also true, so that it would be called tautology, no error in their statements. However, how can we justify the truthfulness of claimed general propositions. The regression and generalized linear models (GLMs) describe the causal relationship between observed variables. ${X}_{1},\dots , {X}_{p}$ is regarded as covariates, which cause the observed response to $Y$. Fisher’s classical likelihood applies to this kind of model with observables only, where fixed parameters represent causal effects of covariates. Via likelihood, the estimation of effects, prediction, various hypotheses testing, and including the absence of effects, have been developed [38–41].

### Generalized Linear Latent Variable Models

Consider that $$\left({x}_{1},{y}_{1}\right),\left({x}_{2},{y}_{2}\right),\dots ,({x}_{n},{y}_{n})$$ are independent observations. Each $${y}_{i}$$ represents response variable and each xi represents a $$p\times 1$$ vector of covariates, that is $${x}_{i}=({x}_{i1},{x}_{i2},\dots ,{x}_{ip})$$ and $$i=\text{1,2},\dots ,n$$ to represent subjects. The joint distribution of $$\left({x}_{i},{ y}_{i}\right)$$ can be written as the conditional distribution of $${x}_{i}$$ given $${y}_{i}$$ and the marginal distribution of $${x}_{i}.$$ We use the notation $$p\left({x}_{i}\right|{y}_{i},\psi )$$ for the conditional distribution of $${y}_{i}$$ given $${x}_{i}$$ and $$p\left({x}_{i}\right|\alpha )$$ for the marginal distribution of $${x}_{i}$$. The complete data density of *(*$${y}_{i},{ x}_{i})$$ for the subject *i* can be written as:

3 $$p\left(\psi,\alpha\right)=p\left(x_i,\psi\right)\ast p\left(\alpha\right).$$

In the conditional distribution $p\left({x}_{i},\psi \right)$, $\psi$ is the $k\times 1$ vector of parameters. In our model, this parameter vector $\psi$ considers regression parameter $\beta$ through $\theta$, zero inflation parameter $\omega$ or $\delta$ and over/under dispersion parameter $\tau$, that is $\psi =(\theta ,\omega ,\tau )$. In the marginal distribution$p\left(\alpha \right)$, $\alpha$ indicates the parameter of covariate distribution. We consider the natural exponential family distribution for the conditional distribution $p\left({x}_{i},\psi \right)$. For the following exponential family distribution, we consider parameter $\theta$.

4 $$p\left(x_i,\theta,\phi\right)=exp\lbrack\frac{y_i\alpha\left(\theta_i\right)-b\left(\theta_i\right)}{d_i\left(\phi\right)}+c\left(y_i,\phi\right)\rbrack$$

Where *y* represents the response variable, $a\left({\theta }_{i}\right)$ is the function of mean parameter $\theta$, and ${d}_{i}\left(\phi \right)$ is the function of scale parameter $\phi$. The parameter $\theta$ is used to link the model to the covariates *x*. Let ${\theta }_{i}$ be a function of the linear predictor ${\eta }_{i}$, that is ${\theta }_{i}=f\left({\eta }_{i}\right)$, where *f* is a monotone differentiable function, known to be the link function and ${\eta }_{i}={x}_{i}^{\text{'}}\beta$. In ${\eta }_{i}, { x}_{i}^{\text{'}}=({x}_{i1},{x}_{i2},\dots ,{x}_{ip})$ is the $p\times 1$ vector of covariates and $\beta =({\beta }_{1},{\beta }_{2},\dots ,{\beta }_{p}{)}^{\text{'}}$ is the $p\times 1$ vector of regression coefficients. If ${\theta }_{i}={\eta }_{i}={x}_{i}^{\text{'}}\beta$, then the link function *f* is said to be a canonical link function. We consider ${d}_{i}\left(\phi \right)=1$ throughout our study, and hence $p\left({y}_{i}\right|{x}_{i},\theta ,\phi )$ would be written as $p\left({y}_{i}\right|{x}_{i},\theta )$ or $\varvec{p}\left({\varvec{y}}_{\varvec{i}}\right|{\varvec{x}}_{\varvec{i}},\varvec{\beta })$. The generalised linear model can be meaningless if many zeros in the information or over/under scattering highlight the information.

In the generalised linear model, covariates can be discrete, ceaseless, or both. We will portray the element in the next barely passages. This paper aims to develop GLLVMs with Laplace approximation and variational approximation based on the above analysis. The GLLVMs are the extended version of GLMs with latent variables. Suppose ${Y}_{ij}$ is the multivariate responses across species with $i=\text{1,2},\dots ,n$ being the observational units, and $j=\text{1,2},\dots ,p$ is the number of species. The expectation of ${Y}_{ij}$ is modelled through the following relationship.

5 $$E\left({Y}_{ij}\right)={\mu }_{ij}={g}^{-1}\left({\eta }_{ij}\right)$$

The ${\varvec{\eta }}_{\varvec{i}\varvec{j}}$ is the linear predictor and $\varvec{g}\left(.\right)$ is a link function. The common link function is given in Table 1.

**Table 1:** The Link function [42]

Link Name	Link	Inverse	1st Derivative
Gaussian/Normal	$\mu$	$\eta$	1
Binomial (Bernoulli: m=1)	$\text{l}\text{n}(\mu /(m-\mu \left)\right)$	$m/(1+\text{exp}\left(-\eta \right))$	$m/\left(\mu \left(m-\mu \right)\right)$
Logit Probit	${{\Phi }}^{-1}(\mu /m)$	$m{\Phi }\left(\eta \right)$	$m/\varphi \left\{{{\Phi }}^{-1}(\mu /m)\right\}$
Log-log	$\text{ln}(-\text{ln}(1-\mu /m))$	$m(1-\text{exp}(-\text{exp}\left(\eta \right)\left)\right)$	$(m\left(1-\mu /m\right)\text{ln}{\left(1-\mu /m\right)}^{-1}$
Poisson *Log	$\text{ln}\left(\mu \right)$	$\text{exp}\left(\eta \right)$	$1/\mu$
Negatif Binomial *NB-C	$\text{l}\text{n}(\mu /(\mu +1/\alpha \left)\right)$	$\text{exp}\left(\eta \right)/\left(\alpha \left(1-\text{exp}\left(\eta \right)\right)\right)$	$1/(\mu +\alpha {\mu }^{2})$
Negatif Binomial *log	$\text{ln}\left(\mu \right)$	$\text{exp}\left(\eta \right)$	$1/\mu$
Gamma *Inverse	$1/\mu$	$1/\eta$	$-1/{\mu }^{2}$
Inverse Gaussian *Inv Quad	$1/{\mu }^{2}$	$1/\sqrt{\eta }$	$-1/{\mu }^{3}$

The linear components of the predictor are similar to that of GLM has the inclusion of random effects listed as follows:

6 $${\eta }_{ij}={\alpha }_{i}+{\beta }_{0j}+{x}_{i}^{\text{'}}{\beta }_{j}+{u}_{i}^{\text{'}}{\lambda }_{j}$$

**Fig. 1:**
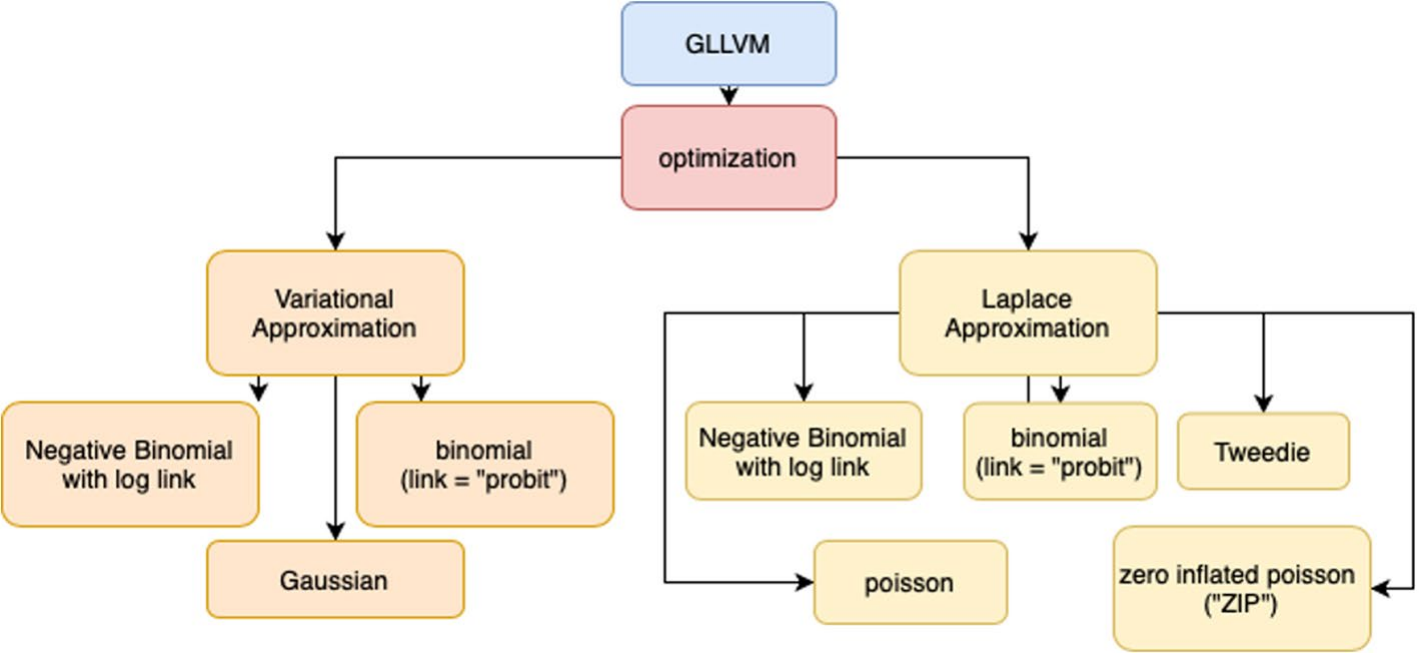
GLLVMs Optimization

The ${\varvec{\alpha }}_{\varvec{i}}$ represents the row effect, ${a\text{n}\text{d} \beta }_{j}$ contains a matrix of the regression coefficient to corresponding independent variables.${x}_{i}^{\text{'}}$ and ${\lambda }_{j}$ are the loading factors or quantities describing the interactions across observation and connecting the unobserved variables to responses [43]. In many papers, the distributional choice of latent variables, ${\varvec{u}}_{\varvec{i}}$ is a normal distribution with mean zero and constant [44–46]. The optimisation in GLLVMs represents in Fig. 1.

**Fig. 2:**
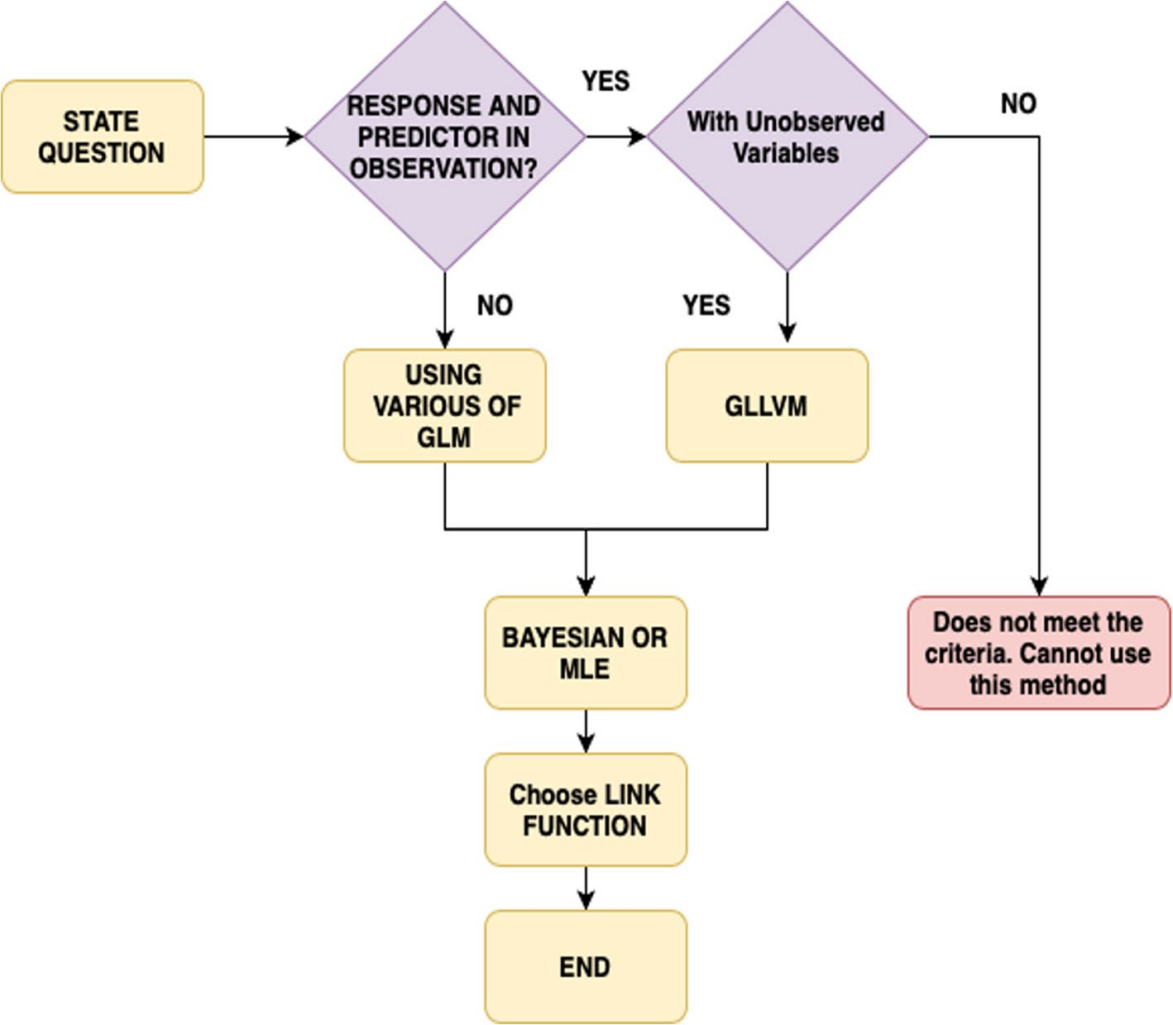
The concept for Choosing Latent GLMs and GLMs Family

The selection of the distribution is another important point in GLM. The distribution preference is dependent on the type of response variable. The mechanism can produce the response and the form of the empirical distribution. For instance, the Bernoulli distribution is the obvious solution for binary responses, whereas the Poisson distribution is also preferred to match the model for counts. The intercept and the slope are also the key parameters to interpret in the standard multivariate regression. The interception is the outcome variable expectation unless the covariates remain zero. The regression coefficients reflect the anticipated variability in the dependent component variables for just a one-unit transition, with the remaining factor being consistent. The parameters may then be represented in Poisson and Negative Binomial methods as in Gaussian because of the log linking function that places variables in the normal log scale [47]. The result is exponential with the parameter through its main sample. This would not resolve the perception problem entirely, as represented in Fig. 2.

### Model Selection

The model selection criteria are statistical tools that identify an “optimal” statistical model from among a set of models. Meanwhile, the set is usually called a set of candidate models. A model is considered [48–50] that is the principle of generalizability to fit the model to describe or predict new data. The purpose of statistical modelling should be to predict new data instead of precisely characterising the actual model that generated the data. On the other hand, the candidate models are significant in analysing the selection criteria.

The criteria can be used Akaike Information Criterion (AIC), Akaike Information Criterion Correction (AICc), and Bayesian Information Criterion (BIC) [51]. Lastly, the selection of models should take generalizability, parsimony, and goodness-of-fit into account. The motivation behind measurable demonstrating ought to anticipate new information rather than unequivocally describe the genuine model that created the information. In Equation (7), *f* as the function of regression, *k* is the dimension of the parameter $\theta ,$ and *n* is the sample size.

7 $$AIC=-2\text{ln}f(y,\widehat\theta)+2k.$$

8 $$BIC=-2\text{ln}f(y,\widehat\theta)+k\text{ln}(n)$$

9 $$AICc=-2k\left[\frac n{n-k-1}\right].$$

However, the researcher leans toward BIC to AIC since BIC may prompt choosing a more closefisted fitted model than AIC. It demonstrates that BIC is steady, yet it is not asymptotically productive. In addition, AICc is helpful in the small dataset.

## High dimension data

In this paper, we use the event count data that occur in the intensive care centre to meet the needs of medical operations. The operations include pushing hospitalised patients for hemodialysis treatment, receiving emergency treatment drugs, transferring specimens, and collecting blood and related services such as respirators, oxygen cylinders, and other equipment or items required for the treatment.

The data used in this research contains the number of events in the intensive care centre to meet the needs of medical operations in Taichung Veterans General Hospital. The specifications are as follows: Emergency Department (ED 1), First Intensive Care Unit (ICU1), Second Intensive Care Unit (ICU2), Respiratory Care Intensive Care Unit (RICU), Surgical Intensive Care Unit (SICU), Subacute Respiratory Care Unit (RCC), Trauma and Neurosurgery Intensive Care Unit (TNCU), Neonatal Intensive Care Unit (NICU). The data are collected from June (33,561 cases), July (31,557 cases), August (35,689 cases), September (34,293 cases), and October (35,310 cases). In total, the matrix dimension is (170,410 × 7). This paper only used eight types of ICU rooms. To get the ICU ordination per room will be transposed to (7 × 170,410). Then the dimension matrix is ​​reduced again to retrieve the total daily occurrence data to get 153 × 7 a matrix. We estimate the latent space’s dimension from the data by using regularised generalised matrix factorisation [52].

**Fig. 3:**
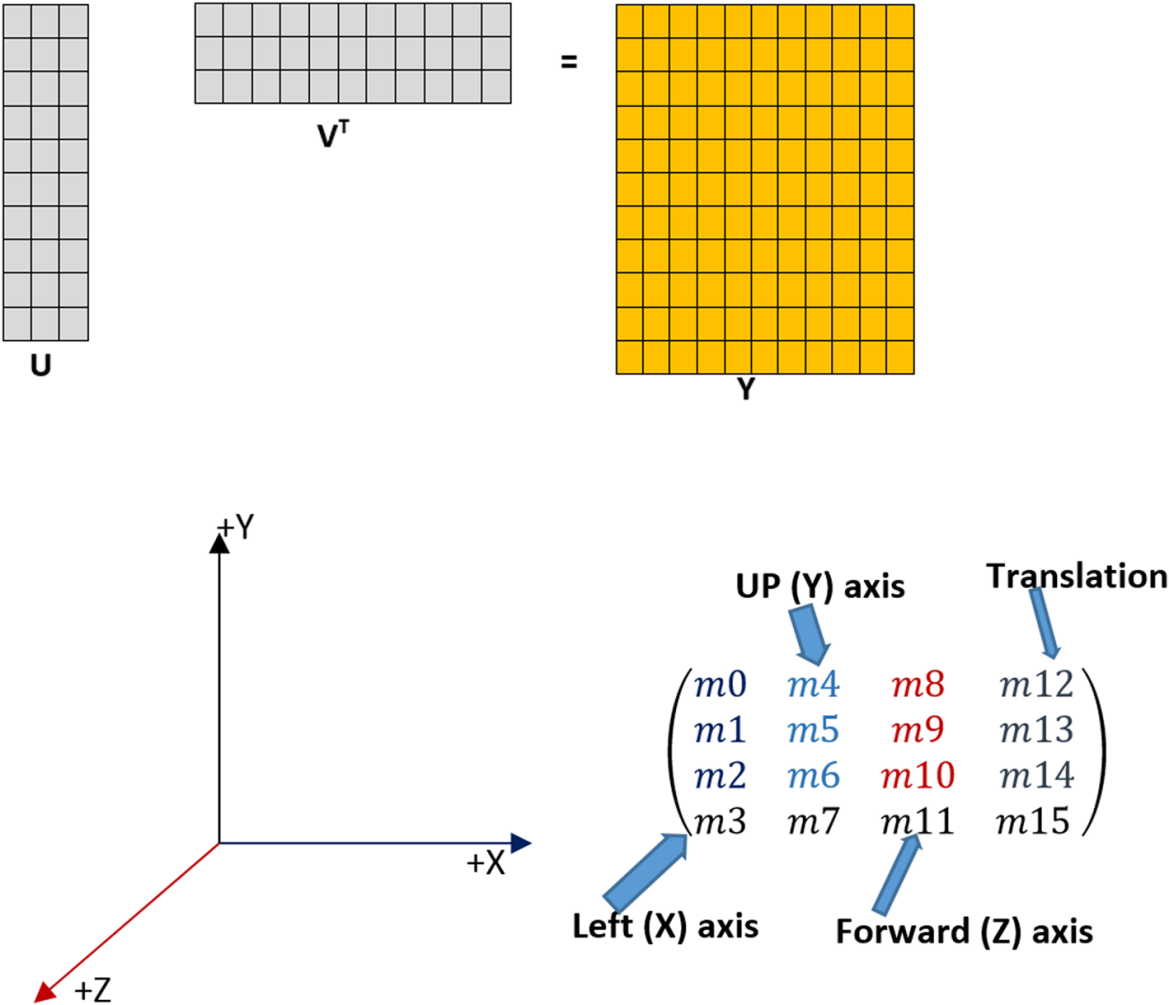
The Example of Projection

Since the dataset is a large size matrix with an observation sufficiently large, the approach would occur error. In the comparison, the method may be unreliable due to round-off errors for too short a break. We placed Newton Raphson (NR) in this analysis to solve these issues. The NR is not intermediate-based and approximates the Hessian matrix-vector product. The pseudo-Hessian matrices have been popularly used [53]. In this study, Fig. 3 represents that the pseudo-Hessian is applied because it has proven to be effective for ${\left[{H}_{diagonal pseudo}\right]}_{k}={v}_{k}^{t} {v}_{k}^{*}$, more instance see: [54].

## Results and discussion

As explained in the previous section, we use the daily data of the number of cases of incentive care rooms. Then, the matrix dimension is quite large. So computation [55] will be calculated on selected distributions such as negative binomials, Poisson, Gaussian, ZIP, and Tweedie. We successfully compared two types of optimisation, including variational approximation and Laplace approximation. Also, we make a comparison with the number of latent variables. Table 2 explains that the best model is the smallest AIC, AICc, and BIC values ​​for the negative distribution of GLLVM-VA and GLLVM-LA binomials. Figure 2a and b have explained that information. In general, VA (1) promises to complete computing time compared to LA (2).

Based on this simulation, we understand that the difference in latent variables does not affect the accuracy results. Besides, the recognisable proof of the estimation model is that it is sufficient for each latent variable. The decision of connection capacity ought to be founded on hypothetical contemplations and model fit. The scope of qualities it creates for the mean ${\mu }_{i}={g}^{-1}$ ${v}_{i},$ can be contemplated when picking the link function. For example, the logit and probit interface capacities are regular when the reaction variable is two-fold. They limit the likelihood ${\mu }_{i}$ within the interval $\left[\text{0,1}\right]$. The other factors consider identifying with the understanding of the relapse parameters [55].

However, utilises an identity link function relates to addictive impacts of the covariates on the mean, and a log link compares to multiplicative effects. Another significant thing in GLLVMs is the decision of the dissemination. The decision of dissemination depends on the kind of reaction variable. The procedure produces the reaction and the state of experimental dispersion. For instance, the undeniable decision is the Bernoulli dissemination for parallel reactions while for counts. In line with this, the Poisson dispersion is regularly picked for fitting the model.

**Table 2:** GLLVMs Performance

Model	LV	Family	Selection Criteria			DF	log-likelihood:	Time Computing
			AIC	AICc	BIC			
GLLVM- VA	1	Negative Binomial	7144.07	7151.123	7207.709	21	-3551.035	00.13,23
	2	Negative Binomial	7309.096	7321.192	7390.918	27	-3627.548	00.05,85
	3	Negative Binomial	7472.096	7489.696	7569.07	32	-3704.048	00.06,11
	1	Poisson	7693.37	7699.733	7753.978	20	-3826.685	00.01,22
	2	Poisson	7693.37	7699.733	7753.978	20	-3826.685	00.03,60
	3	Poisson	7685.202	7695.439	7760.963	25	-3817.601	00.12,20
	1	Gaussian	7260.324	7267.378	7323.964	21	-3609.162	00.01,32
	2	Gaussian	7409.686	7421.782	7491.508	27	-3677.843	00.03,42
	3	Gaussian	7570.224	7587.824	7667.198	32	-3753.112	00.09,10
GLLVM- LA	1	Negative Binomial	6955.922	6962.976	7019.562	21	-3456.961	00.41,30
	2	Negative Binomial	-67622.6	-67622.6	-67622.6	27	3381132785	00.47,11
	1	Poisson	7736.851	7739.895	7779.277	14	-3854.426	00.05,32
	2	Poisson	7387.098	7393.462	7447.707	20	-3673.549	00.44,47
	3	Poisson	7227.31	7237.546	7303.07	25	-3588.655	01.17,90
	1	Gaussian	7107.34	7114.393	7170.979	21	-3532.67	00.01,72
	2	Gaussian	7103.672	7115.768	7185.494	27	-3524.836	00.02,22
	3	Gaussian	7110.665	7128.265	7207.639	32	-3523.333	00.01,88
	1	ZIP	7637.484	7644.538	7701.123	21	-3797.742	00.31.38
	2	ZIP	7326.899	7338.995	7408.721	27	-3636.449	00.59.18
	1	Tweedie	7010.549	7022.645	7092.371	27	-3478.275	45.11,33

**Fig. 4:**
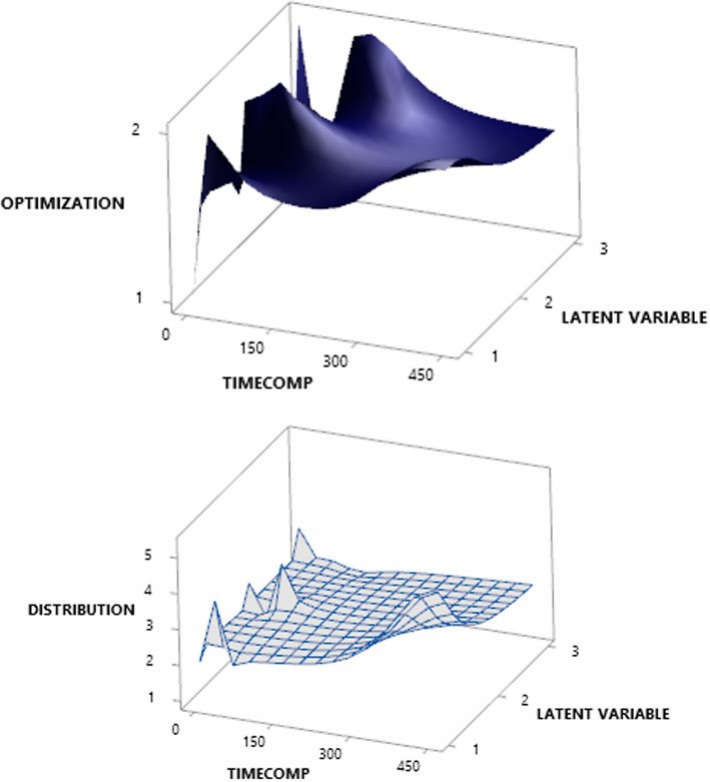
Time Computing Optimization (**A**) and Type of Distribution (**B**)

We use different distributions such as Negative Binomial (1), Poisson (2), Gaussian (3), ZIP (4), and Tweedie (5). As shown in Fig. 4, running a Tweedie distribution will take a very long time. The power parameters are vital to discuss. In tweedy probability density, it cannot be closed form so it is slow to finish computing. To solve this problem, quasi and pseudo-likelihood can be used for Tweedie.

The Tweedie distribution can only be analysed using the Laplace approximation GLLVM. Indeed, a Variational approximation is a Bayesian inference to solve complex statistics. Ormerod [56] gave a more precise explanation about the Variational approximation. On the other hand, Bayesian, along these lines [57] relies upon the researcher’s capacity to compute integrals concerning the posterior distribution. This is a troublesome issue and separated from the conjugate models. The explicit type of the thickness posterior is regularly accessible just to a factor.

$$\pi \left(x\right|{y}_{1},\dots , {y}_{N})\propto {p}_{0}\left(x\right)p({y}_{1},\dots ,{y}_{N}\left|x\right)$$

During the experiment, we compare GLVVMs to PCA, Factor Analysis Extraction Maximum Likelihood, K-Means, Canonical Correlation Analysis, and Global Multidimensional Scaling. However, using *K*-means only uses two groups following the number of groups that have been previously determined. To determine the group members can be done by calculating the minimum distance of the object.

The value obtained in the membership of data at the distance matrix is ​​*0* or *1.* The value *1* is for data allocated to group A while the value 0 is for data allocated to group *B*. In this simulation, we obtained distance centroid (Cluster 1 to Cluster 2 = 24.6436). Table 3 provides variance information (%) of each method. During the experimental studies and the simulation results, GLLVMs promise high variance compared to the other techniques. In line with this, the number of variances can be explained with the latent variable as 98%. Yet, PCA and CCA perform variable reduction via justification and construct a scree plot variance explained (or eigenvalues).

**Table 3:** The Variance

Methods	Variance (%)
PCA	78.5%
Factor Analysis Extraction Maximum Likelihood	75.2%
K-Means 2 cluster	51.070%
*Ours (GLLVM)	98%
Canonical Correlation Analysis	70.2%
Global Multidimensional Scaling	68.5%

**Fig. 5:**
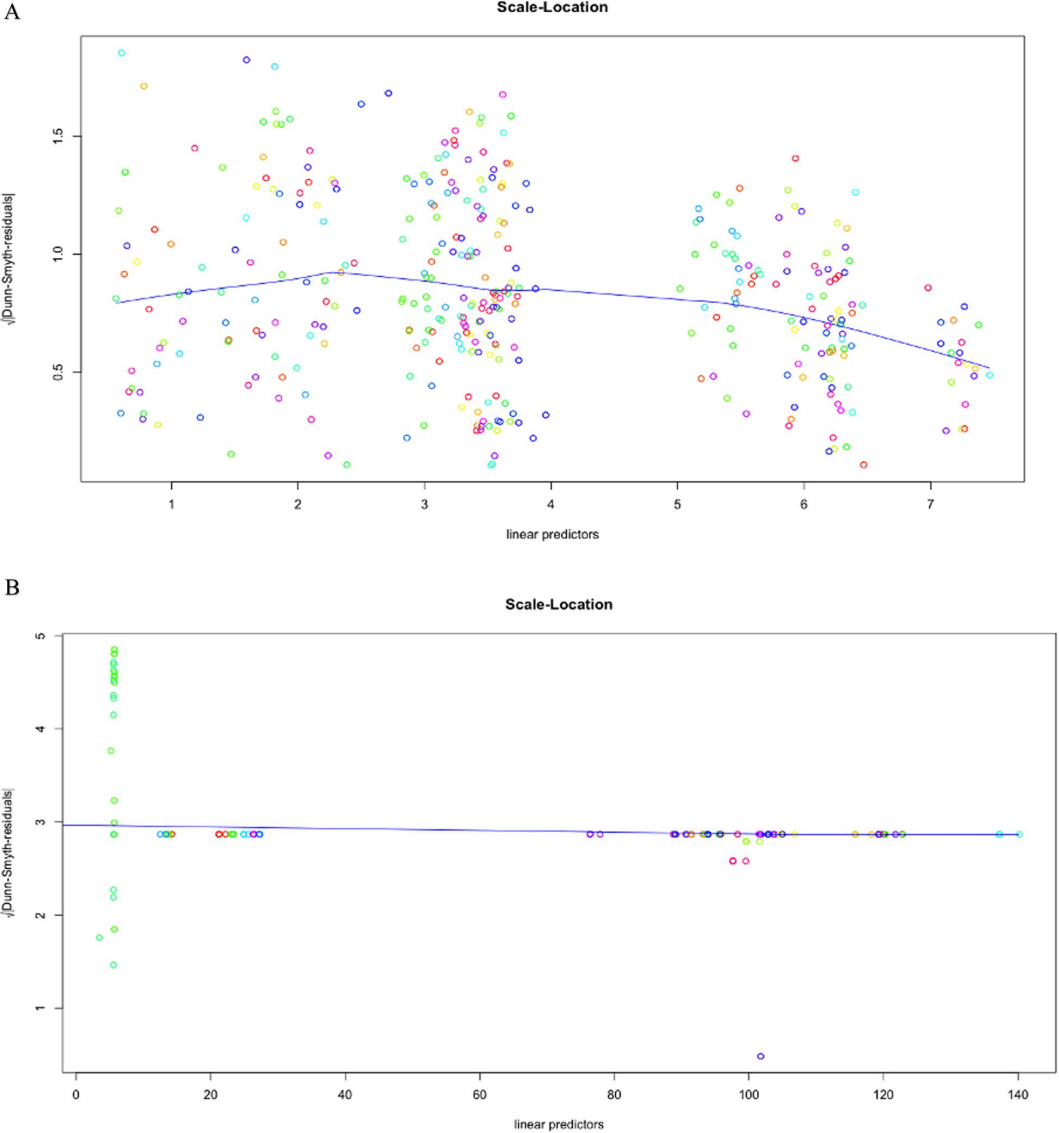
Scale Location GLLVM Negative Binomial with 1 Latent Variable Variational Approximation (**A**) Scale Location GLLVM Negative Binomial with 1 Latent Variable Laplace Approximation (**B**)

**Fig. 6:**
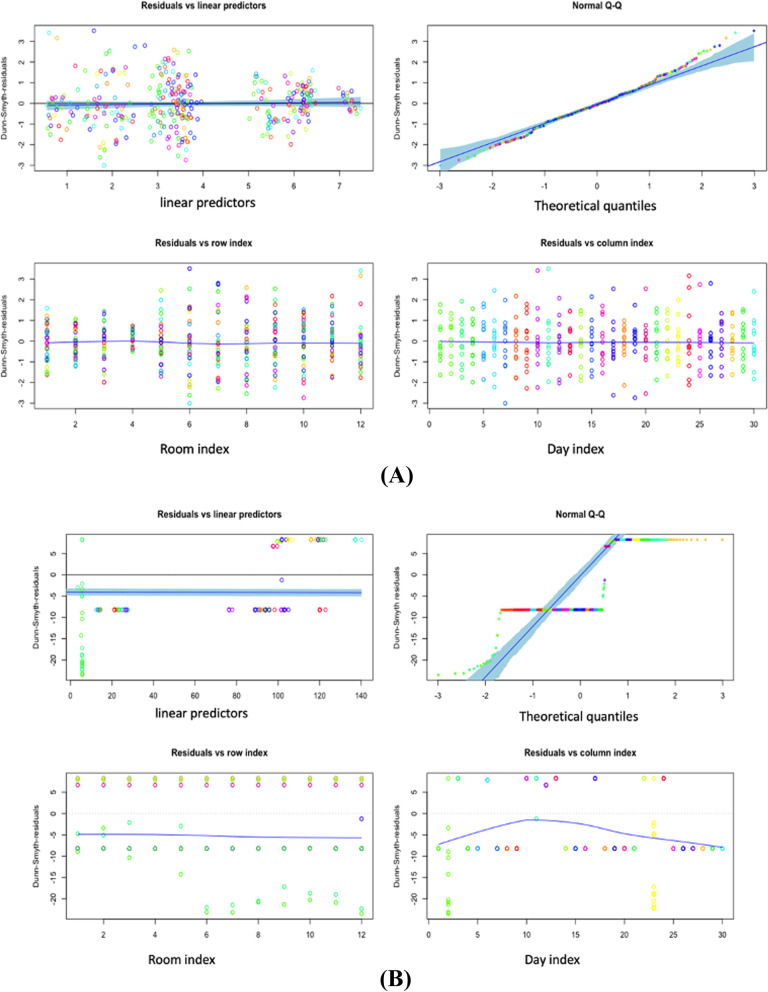
(**A**) GLLVM Negative Binomial with 1 Latent Variable Variational Approximation Residual VS Predictor (**B**) GLLVM Negative Binomial with 1 Latent Variable Laplace Approximation Residual VS Predictor

Meanwhile, two significant methodologies have shown up in measurements, such as approaches dependent on the characterisation of the posterior and approximation. For a differential condition, whose arrangement is not easy work at any rate. The Laplace approximation can tell the arrangement is the inverse Laplace likewise. The underlying conditions are folded into the strategy for the arrangement from the beginning. Nevertheless, with Bayes, we do not have the entirety of the underlying derivatives, so we need to keep some of them around as free parameters. The Laplace, for the most part, is not in nonlinear issues because we do not receive a decent arithmetical condition in return [, 58, 59]. One exception is that the Laplace change of a convolution is only an item helpful [60]. The data matrix is ​​usually a proximity matrix (a matrix with a distance between objects) and includes ordinal data types. This result is robust because the configuration results are obtained from its iteration. However, the process will lose some information due to the reduction in dimensions. The ordination is also helpful in reducing the dimensions of data from several variables. New variables are no longer correlated and have as much information as possible from the original data after getting the best negative binomial model on two different optimisations, Variational approximation and Laplace approximation. It is necessary to find linear predictors with residuals in both models. Figure 5a and b represent scale location. At the beginning of our predictor range, the line starts off horizontal, slopes up to around 2, and then slopes down around 3. In the beginning, contrast with the Laplace approximation, the line is flattened around 2.5 because the residuals for those predictor values are not more spread out. The development of the GLLVM ordination will continue by using a Variational approximation. Assume that it provides speed in computing with accuracy differences that are not significant as the Laplace approximation. Figure 6a and b explain how linear these predictors are at residuals. Then, the normal Quantile-Quantile plot describes the theoretical quantiles following the normal distribution and the points forming a roughly straight line.

**Fig. 7:**
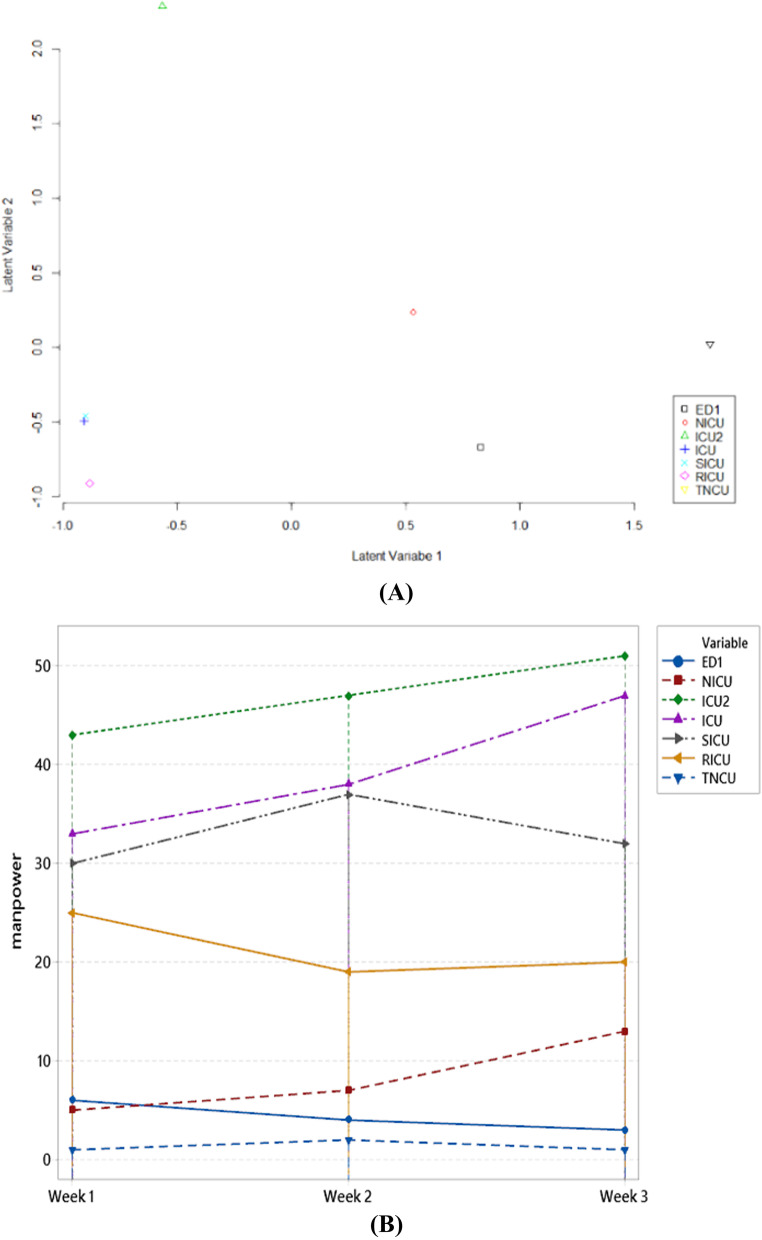
GLLVM Ordination (**A**) and Prediction manpower (**B**)

However, Fig. 7a explains the ordination in seven different room types. It seems so clear that each room has a different ordination. In addition, Fig. 7b represents the number of manpower based on the best model. The type of ICU room requires more manpower than other rooms. Nevertheless, visually ICU and RICU rooms have the same characteristics compared to the others. Overall, the different ordinance is ICU2 room, and separate ordinations are in the RICU room.

**Fig. 8:**
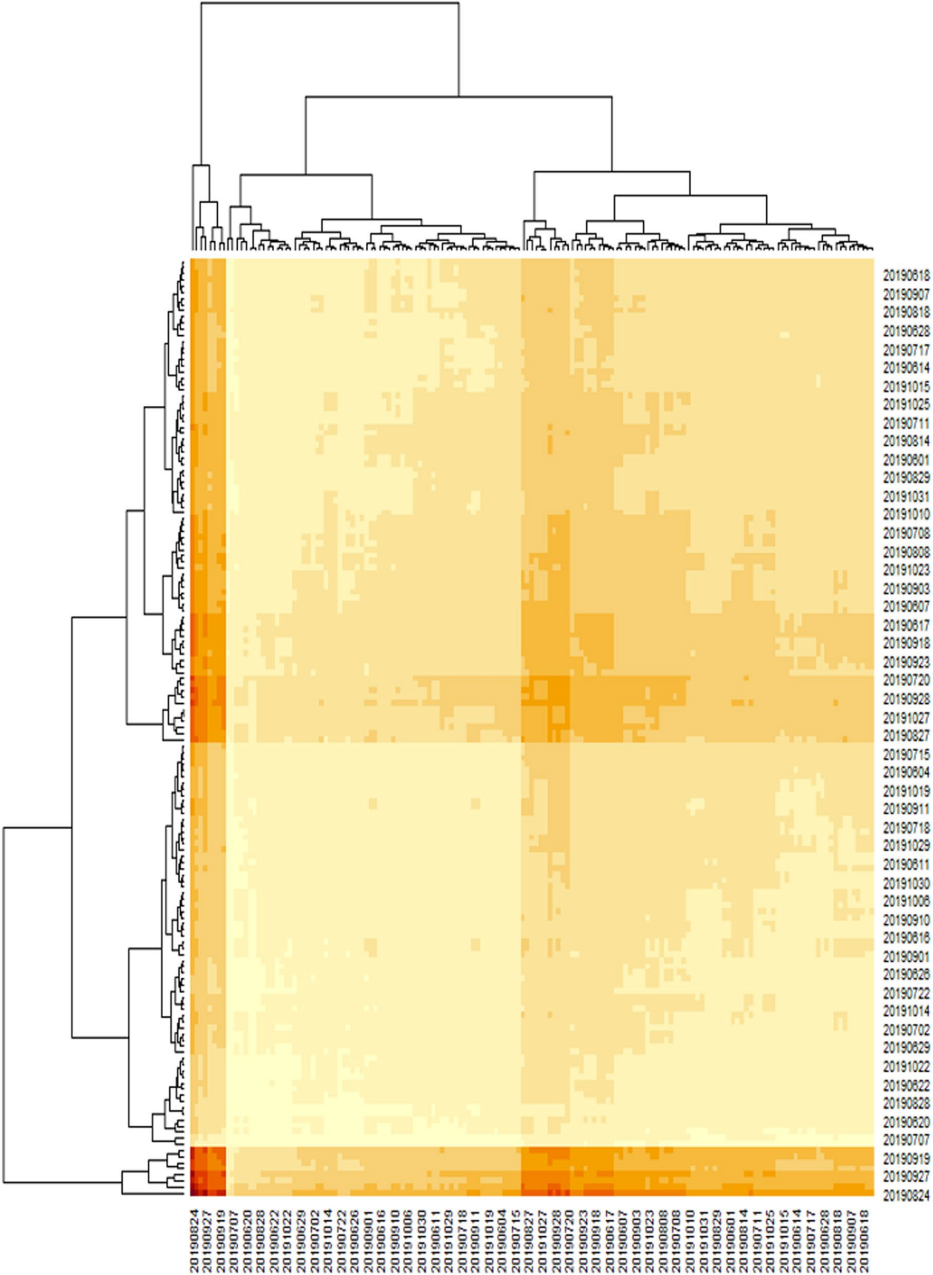
Heatmap event counts

At the same time, the ordinations look similar in ED1 and TNCU rooms, respectively. Figure 8 explains the distribution of frequency of events data in intensive care Units’ rooms if there are several similarities between one day and another. The highest number of cases occurred on Monday and Saturday, and Sunday decreased quite far. If the hospital wants to focus on full service, it might be better to consider the appropriate number of medical staff on a specific day.

The content of the hospital’s transfer staff is to transfer patients to outpatients, wards, inspections, and other units. The transfer methods include leadership, bed and wheelchair push, and the receipt and transfer of medicines, blood, specimens, articles, instruments, and stationery to other units. The outsourcing business of the hospital’s labour service is also to maintain the business activity. The staff is responsible for it, including the ward, medical department, or particular operation unit’s internal labour service. It is fixedly dispatched to the demand unit. Non-medical care services, such as ward replenishment, hand sanitiser, and redemption of infectious devices, medicine ladders, cleaning of dirty clothes, extra isolation clothes, etc., work items will follow the general ward. Moreover, the emergency characteristics of intensive or special controls and departmental treatment units may be different.

Still, their work is non-medical affairs, and responsible for such work belongs to internal staff. This mode’s transmission requirement is mainly related to the relevant operational processes required to treat inpatients. The examinations are X-rays, ultrasound, electrocardiograms, computed tomography (CT) tests or anesthesia visits before the operation of the patient; or pushing inpatients for blood Dialysis treatment, receiving emergency treatment medicines, transferring specimens, and related operations such as respirators, oxygen cylinders, and other equipment or items required for treatments.

**Fig. 9:**
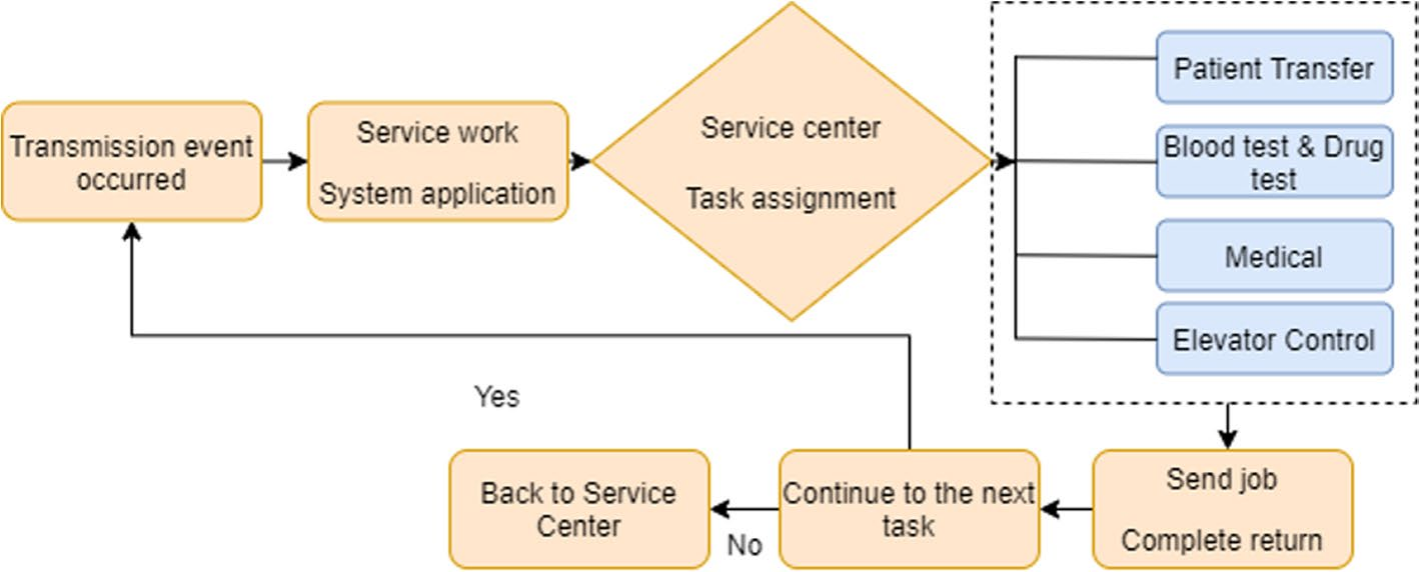
Hospital transfer operation flow

Figure 9 represents the application of the “Hospital Transfer Operating System” by the ward nursing station and the dispatching method which is based on the delivery center. The cases are general, urgent, or scheduled categories. The application event is transmitted to the service center to print the document. The service center dispatches personnel to perform the transmission operation and builds upon the priority of the event transmission or the application sequence. When the transmission staff completes the task, they return to the service center to wait for the next job assignment.

An ICU is an Intensive Care Unit, and CCU, for the most part, represents the Cardiac Care Unit. An emergency is a basic consideration unit that concedes therapeutic and careful patients who are fundamentally sick or harmed. While a Cardiac Care Unit concedes patients with heart issues, it is generally medicinal cardiovascular issues. The respiratory intermediate care unit (RICU) should be practically incorporated with the intensive care hospital room, the general ICU, and the restorative or different wards. These units should be described by higher self-sufficiency than the checking units because of the more elevated level of care [61].

Subsequently, while patients have the intense, the incessant respiratory disappointment of any level of seriousness ought to admit these units for the intubated individuals. Moreover, fundamentally sick patients with weaning issues could be admitted to the RICU. On the other hand, the Surgical Intensive Care Unit provides care for patients who have undergone many critical surgical procedures. SICU will cover Pediatric Vascular, Gastrointestinal Liver, Renal, Renal-Pancreas Transplantation, Orthopedics, Plastics, Otolaryngology, Urology, Thoracic, Surgical Oncology, Oral Maxillo-Facial Obstetrics, and Gynecological Surgery. Management of patient trauma is essential, and this treatment is carried out at the trauma care centre plus (TNCU).

Traumatic patients need airway evaluation and management, respiratory support, bleeding cases, rapid, swift. Patients who come to the emergency unit must go through triage, which evaluates the patient’s condition to determine the emergency level. Patients will be treated according to the category of triage, videlicet, triage one, patients with life-threatening conditions or loss of limb function and require immediate action or intervention with a waiting time of 0 min.

Then, triage two is a patient with a non-life-threatening condition but has a potential threat to limb function and requires prompt medical intervention or action with a waiting time of 0-5 min. Triage three are patients with acute conditions but not urgent (primarily stable). There is no potential to experience worsening and do not require immediate medical intervention or intervention with a waiting time of 5 to 15 min. NICU stands for neonatal intensive care unit, is an intensive care room in the hospital that is explicitly provided for newborns who experience health problems [62].

Generally, babies are placed into the NICU room in the first 24 h after birth. The length of stay in the NICU room varies, depending on each baby’s condition. The more serious the health problem is experienced, the longer they will be in the NICU room. There are many reasons why babies need to be cared for in the NICU room, but they aim to get the child under intensive supervision and care. The NICU room is a sterile area that just no one can enter. Each hospital has a different blueprint regarding the number and hours of parents visiting the NICU room. However, all hospitals must provide soap or hand sanitisers to ensure that visitors are sterile. In general, NICU room conditions are tranquil because they are susceptible to sound and light. The babies in the NICU room are usually in the incubator to keep their body temperature stable. The hospital delivery business is roughly divided into first, patient escort: during the patient’s medical treatment process. The patient is pushed for examination, surgery, kidney dialysis, or related treatment. Additionally, Non-patient is transmission: similar transmission of specimens, drugs, blood, documents, medical records, or medical supplies.

This research transmission business is aimed at front-open patient escort and non-patient transmission. According to the different work attributes of each ward, medical department, or operating unit, the required human resources are divided into four categories, and various types of human resources are ordered according to their complexity or danger.

## Conclusions

This paper successfully performs the simulation of the huge dimensional dataset. The best distribution used is a negative binomial and variational approximation. Interestingly, the choice of the number of latent variables has a significant effect on computational time but not on the model’s accuracy. In general, the more latent used, it will slow down the computing time. The instrument involved the distribution of Tweedie was proven that Tweedie required a very long time compared to other distributions.

Future studies will use different types of distributions, such as extended negative binomials and hurdle distributions. We will compare the distribution zero-inflated Poisson, zero-inflated negative-binomial, beta-binomial, extended Poisson and Tweedie, hurdle, and extended hurdle for further research negative. In many situations, we cannot obtain information about which classes of some observations belong to which group. In this case, we need adaptations to the Variational Approximation and Laplace Approximation.

Posterior probabilities for labeled data do not need to be updated. The other probabilities corresponding to unlabeled data are computed as usual. Discussion, so far, assume that all the classes in the entire data sets are represented in the classes represented in labeled data so that GLLVM is known and model selection is not an issue. However, if the assumption does not hold, several problems arise with initialization on the optimization. One option is to consider only unlabeled information, ignoring the labeled observation. But, by considering and separating the dataset as training and testing, we can check our model is appropriate or not. In many multivariate data sets, some of the variables are highly correlated with others, so that they do not carry much additional information. The elimination of such variables can improve model performance. In Additional file 1: Appendix, We already explain how to calculate a computation of Variational Approximation and Laplace approximation. Otherwise, we may use the regularized log-likelihood function penalized by concern via $-\lambda \sum _{k=1}^{K}\sum _{j=1}^{p}\left|{\mu }_{kj}\right|$ where ${\mu }_{kj}$ is the $j-th$ coordinate of the $k-th$mean vector. Assume the independence of multinomial variables is the response to each spicy, with a *p*-response observed from each individual which can be modeled as a finite products-of-multinomials mixture model.

$$g\left({X}_{jr}; {\pi }_{k},{\rho }_{kjr}|k=\text{1,2},\dots , K,j=\text{1,2},\dots ,p,r=\text{1,2},.,{d}_{j}\right)=\sum _{k=1}^{K}{\pi }_{k}\sum _{j=1}^{p}{\eta }_{j}\prod _{r=1}^{{d}_{j}}\frac{{\rho }_{kjr}^{{x}_{jr}}}{{x}_{jr}!},xjr=\text{0,1},.$$

Future work should extend the basic concept of GLLVMs to Structural Equation Modelling (SEMs) or employ hierarchical likelihood. A frequentist alternative approach is proposed by Lee et al. [42], who termed it as the hierarchical likelihood approach. Hereafter, we use the term *h*-likelihood. Also, it provides a new way of statistical inferences in entire fields of statistical science. Recently, *h*-Likelihood is also commonly used for inferences and the application in big data and machine learning [63]. Therefore, we address the likelihood for fitting SEMs that supports various combinations of different distributions for response variables [48, 64–70]. *h-*likelihood can be defined by the logarithm of the joint density of the response $\varvec{y}$ and the unobserved vectors of random effects $\varvec{v},\varvec{p},$ and $\varvec{q}$ given by

$$h=h\left(\beta ,\gamma ,\delta ,v,p,q;y\right)$$

$$h={\text{log}f}_{\beta ,\gamma ,\delta }\left(y|v,p,q\right)+{\text{log}f}_{\delta }\left(v|q\right)+\text{log}{f}_{\alpha }\left(p\right)+\text{log}{f}_{\xi }\left(q\right)$$

For estimation, we use $h$ for $v,{p}_{v}\left(h\right)$ for $\beta ,{p}_{v,\beta }\left(h\right)$ for $\left(\gamma ,\delta ,p,q\right),{p}_{b,\beta ,\gamma ,p}\left(h\right)$ for $\alpha$ and ${p}_{v,\beta ,\delta ,q}\left(h\right)$ for $\xi$ [71].

## Supplementary Information

**Additional file 1.**

## Data Availability

The data that support the findings of this study are available from the corresponding author upon reasonable request.
