# Correction to: Big data ordination towards intensive care event count cases using fast computing GLLVMS

**DOI:** 10.1186/s12874-022-01602-z

**Published:** 2022-04-18

**Authors:** Rezzy Eko Caraka, Rung-Ching Chen, Su-Wen Huang, Shyue-Yow Chiou, Prana Ugiana Gio, Bens Pardamean

**Affiliations:** 1 Executive Secretariat, National Research and Innovation Agency (BRIN), DKI Jakarta, 10340 Indonesia; 2 Department of Information Management, College of Informatics, Chaoyang University of Technology, Taichung City, 41349 Taiwan; 3 Taichung Veterans General Hospital, Taichung City, 40705 Taiwan; 4 Department of Mathematics, Universitas Sumatera Utara, Medan, 20155 Indonesia; 5 Bioinformatics and Data Science Research Center, Bina Nusantara University, DKI Jakarta, 11480 Indonesia; 6 Computer Science Department, Bina Nusantara University, DKI Jakarta, 11480 Indonesia

**Correction to: BMC Med Res Methodol 22, 77 (2022)**

**https://doi.org/10.1186/s12874-022-01538-4**

Following publication of the original article [1], the authors noticed the incorrect corresponding authors reflected on this article. The corresponding author should be Prof. Rung Ching Chen and Dr. Su Wen Huang. The original article has been updated.
