# Supplementary material for: Big data ordination towards intensive care event count cases using fast computing GLLVMS
Source: BMC Med Res Methodol. 2022 Mar 21;22:77. doi: 10.1186/s12874-022-01538-4 (PMC8939086; doi:10.1186/s12874-022-01538-4)
Supplement: Supplementary file 1 — Additional file 1. [file 12874_2022_1538_MOESM1_ESM.docx]

**APPENDIX**

**A.Employing Variational Approximation**

In the heart of parameter estimation, the data likelihood with a lower bound can be estimated by variational approximation. VA generally has known computational viability and trades the bias. However, if our random vector followed an exponential family, we can write the distribution joint as follows:

$$\boldsymbol{P}\left( \boldsymbol{Z} \right)\boldsymbol{=}\boldsymbol{exp}\left( \boldsymbol{\langle\theta,\phi}\left( \boldsymbol{Z} \right)\boldsymbol{\rangle} \right)\boldsymbol{-A}\left[ \left( \boldsymbol{\theta} \right) \right]$$

(A.1)

Where $\boldsymbol{Z}$ is latent variable X with observed variables Y<x,y> and have inner product between x and y. $\boldsymbol{\theta=}\left( \boldsymbol{\theta}_{\boldsymbol{1}}\boldsymbol{,\ldots,}\boldsymbol{\theta}_{\boldsymbol{n}} \right)^{\boldsymbol{'}}$ s a vector of parameters and $\boldsymbol{\phi}\left( \boldsymbol{X} \right)\boldsymbol{=}\left( \boldsymbol{\phi}_{\boldsymbol{1}}\left( \boldsymbol{X} \right)\boldsymbol{,\ldots,}\boldsymbol{\theta}_{\boldsymbol{n}}\left( \boldsymbol{X} \right) \right)^{\boldsymbol{'}}$ can be defined as the realized value of the variables. However,$\boldsymbol{\theta=log}\int\boldsymbol{exp}\left( \boldsymbol{\langle\theta,\phi(x)\rangle} \right)\boldsymbol{dx}$ is the log partition function and ensures distribution is normalized. The parameters estimation using the method of moments is a generalization of the Gaussian-Poisson model.

$$\boldsymbol{P}\left( \boldsymbol{\theta} \right)\boldsymbol{=N}\left( \boldsymbol{\mu}_{\boldsymbol{1}}\boldsymbol{,}\boldsymbol{\Sigma}_{\boldsymbol{1}} \right)\boldsymbol{, d\in1,\ldots,D}$$

(A.2)

$$\boldsymbol{P}\left( \boldsymbol{X}_{\boldsymbol{1,d}}\boldsymbol{|}\boldsymbol{X}_{\boldsymbol{t,d}}\boldsymbol{\theta} \right)\boldsymbol{=N}\left( {\boldsymbol{X}_{\boldsymbol{t+1,d}}\boldsymbol{|A}}_{\boldsymbol{t}}\boldsymbol{X}_{\boldsymbol{t,d}}\boldsymbol{+}\boldsymbol{b}_{\boldsymbol{t}}\boldsymbol{,}\boldsymbol{v}_{\boldsymbol{t}} \right)\boldsymbol{, d\in1,\ldots,T-1}$$

$$\boldsymbol{P}\left( \boldsymbol{X}_{\boldsymbol{1,d}}\boldsymbol{|}\boldsymbol{X}_{\boldsymbol{t,d}}\boldsymbol{\theta} \right)\boldsymbol{=}\prod_{\boldsymbol{s=1}}^{\boldsymbol{S}} \boldsymbol{P}\left( \boldsymbol{Y}_{\boldsymbol{t,s}\mathbf{,}\boldsymbol{d}}\boldsymbol{|}\boldsymbol{X}_{\boldsymbol{t,k}\left( \boldsymbol{s} \right)\boldsymbol{,d}} \right)$$

$$\boldsymbol{=PO(}\boldsymbol{Y}_{\boldsymbol{t,s,d}}\boldsymbol{|exp (}\boldsymbol{\beta}_{\boldsymbol{0,s}}\boldsymbol{+}\boldsymbol{\beta}_{\boldsymbol{1,s}}\boldsymbol{X}_{\boldsymbol{t,k}\left( \boldsymbol{s} \right)\boldsymbol{,d}}\boldsymbol{)) , s\in1,\ldots,S}$$

(A.3)

The dimensionality of *X* is via the clustering function *k*. By projecting the data, $\boldsymbol{Y}_{\boldsymbol{k}^{\boldsymbol{-1}}\boldsymbol{(}\boldsymbol{s}^{\boldsymbol{'}}\boldsymbol{)}}$ for each cluster $\boldsymbol{s}^{\boldsymbol{'}}$ onto a single dimension of an auxiliary data object $\boldsymbol{Y}_{\boldsymbol{s}^{\boldsymbol{'}}}\boldsymbol{\epsilon}\boldsymbol{N}^{\boldsymbol{T\times1\times D}}\boldsymbol{.}$ The latent process could be fitted to the auxiliary data without encountering this problem:

$${\tilde{\boldsymbol{Y}}}_{\boldsymbol{t,}\boldsymbol{s}^{\boldsymbol{'}}\boldsymbol{,d}}\boldsymbol{=}\boldsymbol{f}_{\boldsymbol{s}^{\boldsymbol{'}}}\left( \boldsymbol{Y}_{\boldsymbol{t-}\boldsymbol{k}^{\boldsymbol{-1}}\left( \boldsymbol{s}^{\boldsymbol{'}} \right)\boldsymbol{,d}} \right)\boldsymbol{,}\boldsymbol{s}^{\boldsymbol{'}}\boldsymbol{\in1,\ldots,M}$$

(A.4)

$$\boldsymbol{\gg}{\tilde{\boldsymbol{\mu}}}_{\boldsymbol{t,}\boldsymbol{s}^{\boldsymbol{'}}}\boldsymbol{=2}{\tilde{\boldsymbol{z}}}_{\boldsymbol{1}}\left( \boldsymbol{t,}\boldsymbol{s}^{\boldsymbol{'}} \right)\boldsymbol{-}\frac{\boldsymbol{1}}{\boldsymbol{2}}{\tilde{\boldsymbol{z}}}_{\boldsymbol{2}}\left( \boldsymbol{t,}\boldsymbol{s}^{\boldsymbol{'}} \right)$$

$${\tilde{\boldsymbol{\sigma}}}_{\boldsymbol{t,}\boldsymbol{s}^{\boldsymbol{'}}}\boldsymbol{=}{\tilde{\boldsymbol{z}}}_{\boldsymbol{2}}\left( \boldsymbol{t,}\boldsymbol{s}^{\boldsymbol{'}} \right)\boldsymbol{-2}{\tilde{\boldsymbol{z}}}_{\boldsymbol{1}}\left( \boldsymbol{t,}\boldsymbol{s}^{\boldsymbol{'}} \right)$$

$${\tilde{\boldsymbol{\sigma}}}_{\left( \boldsymbol{t,}\boldsymbol{s}_{\boldsymbol{1}}^{\boldsymbol{'}} \right)\boldsymbol{,(t,}\boldsymbol{s}_{\boldsymbol{2}}^{\boldsymbol{'}}\boldsymbol{)}}\boldsymbol{=log}\frac{\boldsymbol{E}\left[ {\tilde{\boldsymbol{Y}}}_{\boldsymbol{t,}\boldsymbol{s}_{\boldsymbol{1}}^{\boldsymbol{'}}\boldsymbol{,d}}{\tilde{\boldsymbol{Y}}}_{\boldsymbol{t}^{\boldsymbol{'}}\boldsymbol{,}\boldsymbol{s}_{\boldsymbol{2}}^{\boldsymbol{'}}\boldsymbol{,d}} \right]}{\boldsymbol{E}\left[ {\tilde{\boldsymbol{Y}}}_{\boldsymbol{t,}\boldsymbol{s}_{\boldsymbol{1}}^{\boldsymbol{'}}\boldsymbol{,d}} \right]\boldsymbol{E}\left[ {\tilde{\boldsymbol{Y}}}_{\boldsymbol{t}^{\boldsymbol{'}}\boldsymbol{,}\boldsymbol{s}_{\boldsymbol{2}}^{\boldsymbol{'}}\boldsymbol{,d}} \right]}$$

$$\boldsymbol{t}^{\boldsymbol{'}}\boldsymbol{\in t,t+1,}\left( \boldsymbol{t\neq}\boldsymbol{t}^{\boldsymbol{t}} \right)\boldsymbol{\vee}\left( \boldsymbol{s}_{\boldsymbol{1}}^{\boldsymbol{'}}\boldsymbol{\neq}\boldsymbol{s}_{\boldsymbol{2}}^{\boldsymbol{'}} \right)$$

where $\boldsymbol{f}_{\boldsymbol{s}^{\boldsymbol{'}}}$ are some functions and

$${\tilde{\boldsymbol{z}}}_{\boldsymbol{1}}\left( \boldsymbol{t,}\boldsymbol{s}^{\boldsymbol{'}} \right)\boldsymbol{=log}\left( \boldsymbol{E}\left[ {\tilde{\boldsymbol{Y}}}_{\boldsymbol{t}^{\boldsymbol{'}}\boldsymbol{,}\boldsymbol{s}^{\boldsymbol{'}}\boldsymbol{,d}} \right] \right)$$

(A.5)

$${\tilde{\boldsymbol{z}}}_{\boldsymbol{2}}\left( \boldsymbol{t,}\boldsymbol{s}^{\boldsymbol{'}} \right)\boldsymbol{=log}\left( \boldsymbol{E}\left[ {{\tilde{\boldsymbol{Y}}}^{\boldsymbol{2}}}_{\boldsymbol{t,}\boldsymbol{s}^{\boldsymbol{'}}\boldsymbol{,d}}\boldsymbol{-}{\tilde{\boldsymbol{Y}}}_{\boldsymbol{t,}\boldsymbol{s}^{\boldsymbol{'}}\boldsymbol{,d}} \right] \right)$$

(A.6)

In Eq(A.5) and Eq(A.6) are analogous to $\boldsymbol{z}_{\boldsymbol{1}}\left( \boldsymbol{t,s} \right)\boldsymbol{,}\boldsymbol{z}_{\boldsymbol{2}}\left( \boldsymbol{t,s} \right)\boldsymbol{.}$ In terms of both interpretability and computational convenience, restricting $\boldsymbol{f}_{\boldsymbol{s}^{\boldsymbol{'}}}$ is a linear function of its arguments can be easily justiﬁed, and this approach is taken here. Several choices of function are available in this regard, including cluster averages:

$$\boldsymbol{f}_{\boldsymbol{s}^{\boldsymbol{'}}}\left( \boldsymbol{Y}_{\boldsymbol{t-}\boldsymbol{k}^{\boldsymbol{-1}}\left( \boldsymbol{s}^{\boldsymbol{'}} \right)\boldsymbol{,d}} \right)\boldsymbol{=}\frac{\boldsymbol{1}}{\left| \boldsymbol{k}^{\boldsymbol{-1}}\boldsymbol{(}\boldsymbol{s}^{\boldsymbol{'}}\boldsymbol{)} \right|}\sum_{\boldsymbol{s\in}\boldsymbol{k}^{\boldsymbol{-1}}\boldsymbol{(}\boldsymbol{s}^{\boldsymbol{'}}\boldsymbol{)}} \boldsymbol{Y}_{\boldsymbol{t,s,d}}$$

(A.7)

Then, we can rewrite to random effects:

$$\left( \boldsymbol{s}^{\boldsymbol{'}} \right)\left( \boldsymbol{Y}_{\boldsymbol{t,}\boldsymbol{k}^{\boldsymbol{-1}}\left( \boldsymbol{s}^{\boldsymbol{'}} \right)\boldsymbol{,d}} \right)\boldsymbol{=}\boldsymbol{P}_{\boldsymbol{k}^{\boldsymbol{-1}}\left( \boldsymbol{s}^{\boldsymbol{'}} \right)\boldsymbol{,d}}^{\boldsymbol{RandEffect}}\boldsymbol{Y}_{\boldsymbol{t,}\boldsymbol{k}^{\boldsymbol{-1}}\left( \boldsymbol{s}^{\boldsymbol{'}} \right)\boldsymbol{,d}}$$

(A.8)

$$\boldsymbol{f}_{\boldsymbol{s}^{\boldsymbol{'}}}\left( \boldsymbol{Y}_{\boldsymbol{t,}\boldsymbol{k}^{\boldsymbol{-1}}\left( \boldsymbol{s}^{\boldsymbol{'}} \right)\boldsymbol{,d}} \right)\boldsymbol{=}\boldsymbol{P}_{\boldsymbol{Y}\boldsymbol{k}^{\boldsymbol{-1}}\left( \boldsymbol{s}^{\boldsymbol{'}} \right)}^{\boldsymbol{RandEffect}}\boldsymbol{Y}_{\boldsymbol{t-}\boldsymbol{k}^{\boldsymbol{-1}}\left( \boldsymbol{s}^{\boldsymbol{'}} \right)\boldsymbol{,d}}$$

(A.9)

And a representative dimension projection

$$\boldsymbol{f}_{\boldsymbol{s}^{\boldsymbol{'}}}\left( \boldsymbol{Y}_{\boldsymbol{t,}\boldsymbol{k}^{\boldsymbol{-1}}\left( \boldsymbol{s}^{\boldsymbol{'}} \right)\boldsymbol{,d}} \right)\boldsymbol{=}\boldsymbol{Y}_{\boldsymbol{t,}\boldsymbol{s}^{\boldsymbol{'*}}\boldsymbol{,d}}\boldsymbol{,}\boldsymbol{s}^{\boldsymbol{'}}\boldsymbol{s}$$

(A.10)

One dimension in each random effect is chosen to represent the dimension $\boldsymbol{s}^{\boldsymbol{'*}}$ $\boldsymbol{s}^{\boldsymbol{'}}\boldsymbol{\in}\boldsymbol{k}^{\boldsymbol{-1}}\boldsymbol{(}\boldsymbol{s}^{\boldsymbol{'}}\boldsymbol{)}$ whose average data is over replication. $\frac{\boldsymbol{1}}{\boldsymbol{D}}\sum_{\boldsymbol{d=1}}^{\boldsymbol{D}} \boldsymbol{Y}_{\boldsymbol{s,d}}$was closest in norm to the average data over replication

$$\frac{\boldsymbol{1}}{\boldsymbol{D|}\boldsymbol{k}^{\boldsymbol{-1(}\boldsymbol{s}^{\boldsymbol{'}}\boldsymbol{)}}}\sum_{\boldsymbol{d=1}}^{\boldsymbol{D}} \sum_{\boldsymbol{s}^{\boldsymbol{''}}\boldsymbol{\in}\boldsymbol{k}^{\boldsymbol{-1}}\boldsymbol{(}\boldsymbol{s}^{\boldsymbol{'}}\boldsymbol{)}} \boldsymbol{Y}_{\boldsymbol{s}^{\boldsymbol{''}}\boldsymbol{,s,d}}$$

(A.11)

$$\mathbf{s}^{\mathbf{'*}}\boldsymbol{=}\underset{\boldsymbol{s\in}\boldsymbol{k}^{\boldsymbol{-1}}\boldsymbol{(}\boldsymbol{s}^{\boldsymbol{'}}\boldsymbol{)}}{\mathbf{argmin}} \left\| \frac{\boldsymbol{1}}{\boldsymbol{D}}\sum_{\boldsymbol{d=1}}^{\boldsymbol{D}} \boldsymbol{Y}_{\boldsymbol{:,s,d}}\boldsymbol{-}\frac{\boldsymbol{1}}{\boldsymbol{D|}\boldsymbol{k}^{\boldsymbol{-1}}\left( \boldsymbol{s}^{\boldsymbol{'}} \right)\boldsymbol{|}}\sum_{\boldsymbol{d=1}}^{\boldsymbol{D}} \sum_{\boldsymbol{s}^{\boldsymbol{''}}\boldsymbol{\in}\boldsymbol{k}^{\boldsymbol{-1}}\boldsymbol{(}\boldsymbol{s}^{\boldsymbol{'}}\boldsymbol{)}} \boldsymbol{Y}_{\boldsymbol{:.}\boldsymbol{s}^{\boldsymbol{''}}\boldsymbol{,d}} \right\|$$

(A.12)

Also, if more than one dimension in $\boldsymbol{k}^{\boldsymbol{-1}}\left( \boldsymbol{s}^{\boldsymbol{'}} \right)$ minimizes, one of the norm of them can be chosen arbitrarily. Once a representative dimension for each cluster $\boldsymbol{s}^{\boldsymbol{'}}\boldsymbol{\in1,\ldots, M}$ has been chosen, sample moments from the data can be used to approximate the expected values and to estimate the parameters of the latent process.

**B. Employing Laplace Approximation**

The marginal density of the manifest variables defined in the Eq can be rewritten as Eq (B.1).

$$\boldsymbol{L}\left( \boldsymbol{\omega,\phi} \right)\boldsymbol{=}\int\boldsymbol{exp}\left( \boldsymbol{p}\sum_{\boldsymbol{i=1}}^{\boldsymbol{n}} \frac{\boldsymbol{1}}{\boldsymbol{p}}\left[ \frac{\boldsymbol{y}_{\boldsymbol{ij}}\boldsymbol{\theta}_{\boldsymbol{ij}}\boldsymbol{-b}\boldsymbol{(\theta}_{\boldsymbol{ij}}\boldsymbol{)}}{\boldsymbol{\phi}_{\boldsymbol{j}}}\boldsymbol{+c}\left( \boldsymbol{y}_{\boldsymbol{ij}}\boldsymbol{,}\boldsymbol{\phi}_{\boldsymbol{j}} \right) \right]\boldsymbol{h(}\boldsymbol{u}_{\boldsymbol{i}}\boldsymbol{)} \right)\boldsymbol{d}\boldsymbol{u}_{\boldsymbol{i}}$$

(B.1)

Since we assume the latent variable $\boldsymbol{u}_{\boldsymbol{i}}$ to follow a standard normal distribution, such that

$$\boldsymbol{h(u}_{\boldsymbol{i}}\boldsymbol{)=}\frac{\boldsymbol{1}}{\left( \boldsymbol{2}\boldsymbol{\pi} \right)^{\frac{\boldsymbol{q}}{\boldsymbol{2}}}}\boldsymbol{exp (}\frac{\boldsymbol{-}\boldsymbol{u}_{\boldsymbol{i}}^{\boldsymbol{'}}\boldsymbol{u}_{\boldsymbol{i}}}{\boldsymbol{2}}\boldsymbol{)}$$

(B.2)

$$\boldsymbol{log}\boldsymbol{h(u}_{\boldsymbol{i}}\boldsymbol{)=}\frac{\boldsymbol{-}\boldsymbol{u}_{\boldsymbol{i}}^{\boldsymbol{'}}\boldsymbol{u}_{\boldsymbol{i}}}{\boldsymbol{2}}\boldsymbol{-}\frac{\boldsymbol{q}}{\boldsymbol{2}}\boldsymbol{log (2}\boldsymbol{\pi)}$$

(B.3)

Then, substituting Eq (B.4) above we have

$$\boldsymbol{L}\left( \boldsymbol{\omega,\phi} \right)\boldsymbol{=}\boldsymbol{\int}\boldsymbol{exp}\left( \boldsymbol{p}\sum_{\boldsymbol{i=1}}^{\boldsymbol{n}} \frac{\boldsymbol{1}}{\boldsymbol{p}}\left[ \frac{\boldsymbol{y}_{\boldsymbol{ij}}\boldsymbol{\theta}_{\boldsymbol{ij}}\boldsymbol{-b}\boldsymbol{(\theta}_{\boldsymbol{ij}}\boldsymbol{)}}{\boldsymbol{\phi}_{\boldsymbol{j}}}\boldsymbol{+c}\left( \boldsymbol{y}_{\boldsymbol{ij}}\boldsymbol{,}\boldsymbol{\phi}_{\boldsymbol{j}} \right) \right]\frac{\boldsymbol{-}\boldsymbol{u}_{\boldsymbol{i}}^{\boldsymbol{'}}\boldsymbol{u}_{\boldsymbol{i}}}{\boldsymbol{2}}\boldsymbol{-}\frac{\boldsymbol{q}}{\boldsymbol{2}}\boldsymbol{log (2}\boldsymbol{\pi)} \right)\boldsymbol{d}\boldsymbol{u}_{\boldsymbol{i}}$$

(B.4)

Suppose we denote

$$\boldsymbol{Q}\left( \boldsymbol{y}_{\boldsymbol{i}}\boldsymbol{,}\boldsymbol{u}_{\boldsymbol{i}}\boldsymbol{,\omega,\phi} \right)\boldsymbol{=}\sum_{\boldsymbol{i=1}}^{\boldsymbol{n}} \frac{\boldsymbol{1}}{\boldsymbol{p}}\left[ \frac{\boldsymbol{y}_{\boldsymbol{ij}}\boldsymbol{\theta}_{\boldsymbol{ij}}\boldsymbol{-b}\boldsymbol{(\theta}_{\boldsymbol{ij}}\boldsymbol{)}}{\boldsymbol{\phi}_{\boldsymbol{j}}}\boldsymbol{+c}\left( \boldsymbol{y}_{\boldsymbol{ij}}\boldsymbol{,}\boldsymbol{\phi}_{\boldsymbol{j}} \right) \right]\frac{\boldsymbol{-}\boldsymbol{u}_{\boldsymbol{i}}^{\boldsymbol{'}}\boldsymbol{u}_{\boldsymbol{i}}}{\boldsymbol{2}}\boldsymbol{-}\frac{\boldsymbol{q}}{\boldsymbol{2}}\boldsymbol{log}\left( \boldsymbol{2}\boldsymbol{\pi} \right)\boldsymbol{.}$$

(B.5)

The particular case of counts data can be modeled using negative binomial distribution [48], [52]. Let $\boldsymbol{y}_{\boldsymbol{ij}}\boldsymbol{|}\boldsymbol{u}_{\boldsymbol{i}}$ have a negative binomial with mean $\boldsymbol{\mu}$ and variance $\boldsymbol{\mu+\alpha}\boldsymbol{\mu}^{\boldsymbol{2}}$. Using the log link function, we have the same relationship between $\boldsymbol{\mu}$ and the term$\boldsymbol{v}_{\boldsymbol{ij}}$ as the Poisson model. The scale parameters are $\boldsymbol{\phi=1,b}\left( \boldsymbol{\theta} \right)\boldsymbol{=}\frac{\boldsymbol{1}}{\boldsymbol{\alpha}}\boldsymbol{log (1+\alpha\mu)}$ and $\boldsymbol{c}\left( \boldsymbol{x,\phi} \right)\boldsymbol{=}\left( \boldsymbol{log}\frac{\boldsymbol{\Gamma}\left( \boldsymbol{y+1/\alpha} \right)}{\boldsymbol{\Gamma}\left( \frac{\boldsymbol{1}}{\boldsymbol{\alpha}} \right)\boldsymbol{\Gamma(y+1)}} \right)$. The conditional distribution on $\boldsymbol{u}_{\boldsymbol{i}}$ is given in Eq (B.6).

$$\boldsymbol{g}_{\boldsymbol{j}}\left( \boldsymbol{u}_{\boldsymbol{i}} \right)\boldsymbol{=exp}\left[ \boldsymbol{y}_{\boldsymbol{ij}}\boldsymbol{log} \frac{\boldsymbol{\alpha}_{\boldsymbol{j}}\boldsymbol{xp (}\boldsymbol{v}_{\boldsymbol{ij}}\boldsymbol{)}}{\boldsymbol{1+}\boldsymbol{\alpha}_{\boldsymbol{j}}\boldsymbol{exp (}\boldsymbol{v}_{\boldsymbol{ij}}\boldsymbol{)}}\boldsymbol{-}\frac{\boldsymbol{1}}{\boldsymbol{\alpha}_{\boldsymbol{j}}}\boldsymbol{log (1+}\boldsymbol{\alpha}_{\boldsymbol{j}}\boldsymbol{exp}\boldsymbol{v}_{\boldsymbol{ij}}\boldsymbol{))+ log}\frac{\boldsymbol{\Gamma}\left( \boldsymbol{y}_{\boldsymbol{ij}}\boldsymbol{+1/}\boldsymbol{\alpha}_{\boldsymbol{j}} \right)}{\boldsymbol{\Gamma}\left( \frac{\boldsymbol{1}}{\boldsymbol{\alpha}_{\boldsymbol{j}}} \right)\boldsymbol{\Gamma(}\boldsymbol{y}_{\boldsymbol{ij}}\boldsymbol{+1)}} \right]$$

(B.6)

The log-likelihood function for negative binomial responses is

$$\boldsymbol{l}\left( \boldsymbol{\omega,\phi} \right)\boldsymbol{=}\sum_{\boldsymbol{i=1}}^{\boldsymbol{n}} \left( \boldsymbol{-}\frac{\boldsymbol{1}}{\boldsymbol{2}}\boldsymbol{log}\boldsymbol{det}\left( \boldsymbol{H}\left( \boldsymbol{\omega,\phi} \right) \right)\boldsymbol{+}\sum_{\boldsymbol{j=1}}^{\boldsymbol{p}} \boldsymbol{[}\boldsymbol{y}_{\boldsymbol{ij}}\boldsymbol{log} \frac{\boldsymbol{\alpha}_{\boldsymbol{j}}\boldsymbol{exp}\boldsymbol{(}\boldsymbol{v}_{\boldsymbol{ij}}\boldsymbol{)}}{\boldsymbol{1+}\boldsymbol{\alpha}_{\boldsymbol{j}}\boldsymbol{exp}\boldsymbol{(}\boldsymbol{v}_{\boldsymbol{ij}}\boldsymbol{)}} \right)\boldsymbol{-}\frac{\boldsymbol{1}}{\boldsymbol{\alpha}_{\boldsymbol{j}}}\boldsymbol{log (1+}\boldsymbol{\alpha}_{\boldsymbol{j}}\boldsymbol{exp} \boldsymbol{v}_{\boldsymbol{ij}}\boldsymbol{))+ log}\frac{\boldsymbol{\Gamma}\left( \boldsymbol{y+}\frac{\boldsymbol{1}}{\boldsymbol{\alpha}} \right)}{\boldsymbol{\Gamma}\left( \frac{\boldsymbol{1}}{\boldsymbol{\alpha}} \right)\boldsymbol{\Gamma}\left( \boldsymbol{y+1} \right)}\boldsymbol{]-}\frac{\boldsymbol{u}_{\boldsymbol{i}}^{\boldsymbol{'}}\boldsymbol{u}_{\boldsymbol{i}}}{\boldsymbol{2}}$$

(B.7)

where

$$\boldsymbol{H}\left( \boldsymbol{\omega,\phi} \right)\boldsymbol{=}\sum_{\boldsymbol{j=1}}^{\boldsymbol{p}} \frac{\boldsymbol{exp (}\boldsymbol{v}_{\boldsymbol{ij}}\boldsymbol{)}}{\left( \boldsymbol{1+}\boldsymbol{\alpha}_{\boldsymbol{j}}\boldsymbol{exp (}\boldsymbol{v}_{\boldsymbol{ij}}\boldsymbol{)} \right)^{\boldsymbol{2}}}\boldsymbol{\lambda}_{\boldsymbol{j}}^{\boldsymbol{T}}\boldsymbol{\lambda}_{\boldsymbol{j}}\boldsymbol{+}\boldsymbol{I}_{\boldsymbol{q}}$$

(B.8)

And $\hat{\boldsymbol{u}_{\boldsymbol{i}}}$ is the solution of

$$\hat{\boldsymbol{u}_{\boldsymbol{i}}}\boldsymbol{=}\sum_{\boldsymbol{j=1}}^{\boldsymbol{p}} \left[ \boldsymbol{y}_{\boldsymbol{ij}}\frac{\left( \boldsymbol{1+}\boldsymbol{\alpha}_{\boldsymbol{j}}\boldsymbol{exp (}\boldsymbol{v}_{\boldsymbol{ij}}\boldsymbol{)} \right)^{\boldsymbol{2}}}{\boldsymbol{exp}\boldsymbol{(}\boldsymbol{v}_{\boldsymbol{ij}}\boldsymbol{)}}\boldsymbol{+}\frac{\boldsymbol{exp}\boldsymbol{(}\boldsymbol{v}_{\boldsymbol{ij}}\boldsymbol{)}}{\boldsymbol{1+}\boldsymbol{\alpha}_{\boldsymbol{j}}\boldsymbol{exp}\boldsymbol{(}\boldsymbol{v}_{\boldsymbol{ij}}\boldsymbol{)}} \right]\boldsymbol{\lambda}_{\boldsymbol{j}}$$

(B.9)

**C. Posterior computation towards Laplace approximation**

For the Laplace distribution case, we can derive the posterior distribution of $\boldsymbol{\eta}_{\boldsymbol{ni}}$ as below:

$$\boldsymbol{\pi(}\boldsymbol{\eta}_{\boldsymbol{ni}}\boldsymbol{|}\boldsymbol{t}_{\boldsymbol{ni}}\boldsymbol{,}\boldsymbol{\pi}_{\boldsymbol{0}}\boldsymbol{,\sigma,b)}$$

$$\boldsymbol{\propto f}\left( \boldsymbol{t}_{\boldsymbol{ni}} | \boldsymbol{\eta}_{\boldsymbol{ni}}\boldsymbol{,\sigma,b} \right)$$

$$\boldsymbol{=}\frac{\sqrt{\boldsymbol{n}}}{\sqrt{\boldsymbol{2}\boldsymbol{\pi\sigma}}}\exp\left[ \boldsymbol{-}\frac{\boldsymbol{n}\left( \boldsymbol{t}_{\boldsymbol{ni}}\boldsymbol{-}\boldsymbol{\eta}_{\boldsymbol{ni}} \right)^{\boldsymbol{2}}}{\boldsymbol{2}\boldsymbol{\sigma}^{\boldsymbol{2}}} \right]\boldsymbol{\times}\left( \boldsymbol{\pi}_{\boldsymbol{0}}\boldsymbol{I}\left( \boldsymbol{\eta}_{\boldsymbol{ni}}\boldsymbol{=0} \right)\boldsymbol{+}\left( \boldsymbol{1-}\boldsymbol{\pi}_{\boldsymbol{0}} \right)\frac{\boldsymbol{1}}{\boldsymbol{2}\boldsymbol{b}}\exp\left[ \boldsymbol{-}\frac{\left| \boldsymbol{\eta}_{\boldsymbol{ni}} \right|}{\boldsymbol{b}} \right]\boldsymbol{I}\left( \boldsymbol{\eta}_{\boldsymbol{ni}}\boldsymbol{\neq0} \right) \right)$$

$$\boldsymbol{=}\boldsymbol{\pi}_{\boldsymbol{0}}\frac{\sqrt{\boldsymbol{n}}}{\sqrt{\boldsymbol{2}\boldsymbol{\pi\sigma}}}\exp\left[ \boldsymbol{-}\frac{\boldsymbol{n}\boldsymbol{t}_{\boldsymbol{ni}}^{\boldsymbol{2}}}{\boldsymbol{2}\boldsymbol{\sigma}^{\boldsymbol{2}}} \right]\boldsymbol{I}\left( \boldsymbol{\eta}_{\boldsymbol{ni}}\boldsymbol{=0} \right)$$

$$\boldsymbol{+}\left( \boldsymbol{1-}\boldsymbol{\pi}_{\boldsymbol{0}} \right)\frac{\sqrt{\boldsymbol{n}}}{\sqrt{\boldsymbol{2}\boldsymbol{\pi\sigma}}}\frac{\boldsymbol{1}}{\boldsymbol{2}\boldsymbol{b}}\exp\left[ \boldsymbol{-}\frac{\boldsymbol{n}\left( \boldsymbol{t}_{\boldsymbol{ni}}\boldsymbol{-}\boldsymbol{\eta}_{\boldsymbol{ni}} \right)^{\boldsymbol{2}}}{\boldsymbol{2}\boldsymbol{\sigma}^{\boldsymbol{2}}}\boldsymbol{-}\frac{\left| \boldsymbol{\eta}_{\boldsymbol{ni}} \right|}{\boldsymbol{b}} \right]\boldsymbol{I}\left( \boldsymbol{\eta}_{\boldsymbol{ni}}\boldsymbol{\neq0} \right)$$

(C.1)

$$\boldsymbol{=}\boldsymbol{\pi}_{\boldsymbol{0}}\frac{\sqrt{\boldsymbol{n}}}{\sqrt{\boldsymbol{2}\boldsymbol{\pi\sigma}}}\exp\left[ \boldsymbol{-}\frac{\boldsymbol{n}\boldsymbol{t}_{\boldsymbol{ni}}^{\boldsymbol{2}}}{\boldsymbol{2}\boldsymbol{\sigma}^{\boldsymbol{2}}} \right]\boldsymbol{I}\left( \boldsymbol{\eta}_{\boldsymbol{ni}}\boldsymbol{=0} \right)$$

$$\boldsymbol{+}\left( \boldsymbol{1-}\boldsymbol{\pi}_{\boldsymbol{0}} \right)\frac{\sqrt{\boldsymbol{n}}}{\sqrt{\boldsymbol{2}\boldsymbol{\pi\sigma}}}\frac{\boldsymbol{1}}{\boldsymbol{2}\boldsymbol{b}}\exp\left[ \boldsymbol{-}\frac{\boldsymbol{n}\left( \boldsymbol{t}_{\boldsymbol{ni}}\boldsymbol{-}\boldsymbol{\eta}_{\boldsymbol{ni}} \right)^{\boldsymbol{2}}}{\boldsymbol{2}\boldsymbol{\sigma}^{\boldsymbol{2}}}\boldsymbol{-}\frac{\left| \boldsymbol{\eta}_{\boldsymbol{ni}} \right|}{\boldsymbol{b}} \right]\boldsymbol{I}\left( \boldsymbol{\eta}_{\boldsymbol{ni}}\boldsymbol{<0} \right)$$

$$\boldsymbol{+}\left( \boldsymbol{1-}\boldsymbol{\pi}_{\boldsymbol{0}} \right)\frac{\sqrt{\boldsymbol{n}}}{\sqrt{\boldsymbol{2}\boldsymbol{\pi\sigma}}}\frac{\boldsymbol{1}}{\boldsymbol{2}\boldsymbol{b}}\exp\left[ \boldsymbol{-}\frac{\boldsymbol{n}\left( \boldsymbol{t}_{\boldsymbol{ni}}\boldsymbol{-}\boldsymbol{\eta}_{\boldsymbol{ni}} \right)^{\boldsymbol{2}}}{\boldsymbol{2}\boldsymbol{\sigma}^{\boldsymbol{2}}}\boldsymbol{-}\frac{\left| \boldsymbol{\eta}_{\boldsymbol{ni}} \right|}{\boldsymbol{b}} \right]\boldsymbol{I}\left( \boldsymbol{\eta}_{\boldsymbol{ni}}\boldsymbol{>0} \right)$$

(C.2)

$$\boldsymbol{=}\boldsymbol{\pi}_{\boldsymbol{0}}\frac{\sqrt{\boldsymbol{n}}}{\sqrt{\boldsymbol{2}\boldsymbol{\pi\sigma}}}\exp\left[ \boldsymbol{-}\frac{\boldsymbol{n}\boldsymbol{t}_{\boldsymbol{ni}}^{\boldsymbol{2}}}{\boldsymbol{2}\boldsymbol{\sigma}^{\boldsymbol{2}}} \right]\boldsymbol{I}\left( \boldsymbol{\eta}_{\boldsymbol{ni}}\boldsymbol{=0} \right)$$

$$\boldsymbol{+}\left( \boldsymbol{1-}\boldsymbol{\pi}_{\boldsymbol{0}} \right)\frac{\boldsymbol{1}}{\boldsymbol{2}\boldsymbol{b}}\exp\left[ \frac{\boldsymbol{t}_{\boldsymbol{ni}}}{\boldsymbol{b}}\boldsymbol{+}\frac{\boldsymbol{\sigma}^{\boldsymbol{2}}}{\boldsymbol{2}\boldsymbol{n}\boldsymbol{b}^{\boldsymbol{2}}} \right]\frac{\sqrt{\boldsymbol{n}}}{\sqrt{\boldsymbol{2}\boldsymbol{\pi\sigma}}}\exp\left[ \boldsymbol{-}\frac{\boldsymbol{n}}{\boldsymbol{2}\boldsymbol{\sigma}^{\boldsymbol{2}}}\left( \boldsymbol{\eta}_{\boldsymbol{ni}}\boldsymbol{-}\left( \boldsymbol{t}_{\boldsymbol{ni}}\boldsymbol{+}\frac{\boldsymbol{\sigma}^{\boldsymbol{2}}}{\boldsymbol{nb}} \right) \right)^{\boldsymbol{2}} \right]\boldsymbol{I}\left( \boldsymbol{\eta}_{\boldsymbol{ni}}\boldsymbol{<0} \right)$$

$$\boldsymbol{+}\left( \boldsymbol{1-}\boldsymbol{\pi}_{\boldsymbol{0}} \right)\frac{\boldsymbol{1}}{\boldsymbol{2}\boldsymbol{b}}\exp\left[ \boldsymbol{-}\frac{\boldsymbol{t}_{\boldsymbol{ni}}}{\boldsymbol{b}}\boldsymbol{+}\frac{\boldsymbol{\sigma}^{\boldsymbol{2}}}{\boldsymbol{2}\boldsymbol{n}\boldsymbol{b}^{\boldsymbol{2}}} \right]\frac{\sqrt{\boldsymbol{n}}}{\sqrt{\boldsymbol{2}\boldsymbol{\pi\sigma}}}\exp\left[ \boldsymbol{-}\frac{\boldsymbol{n}}{\boldsymbol{2}\boldsymbol{\sigma}^{\boldsymbol{2}}}\left( \boldsymbol{\eta}_{\boldsymbol{ni}}\boldsymbol{-}\left( \boldsymbol{t}_{\boldsymbol{ni}}\boldsymbol{-}\frac{\boldsymbol{\sigma}^{\boldsymbol{2}}}{\boldsymbol{nb}} \right) \right)^{\boldsymbol{2}} \right]\boldsymbol{I}\left( \boldsymbol{\eta}_{\boldsymbol{ni}}\boldsymbol{<0} \right)$$

(C.3)

As seen from above, the posterior distribution is divided into three parts. For the first term, we draw 0 for $\boldsymbol{\eta}_{\boldsymbol{ni}}$ with weight obtained from the first term. For the second and third terms, their posterior distributions are distributed as a truncated normal distribution. When $\boldsymbol{\eta}_{\boldsymbol{ni}}$ is less than 0, the posterior for $\boldsymbol{\eta}_{\boldsymbol{ni}}$is drawn from a truncated normal distribution with mean $\boldsymbol{t}_{\boldsymbol{ni}}\boldsymbol{\sigma}^{\boldsymbol{2}}$ and $\boldsymbol{nb}$ variance $\boldsymbol{\sigma}^{\boldsymbol{2}}$.
